# Splice-site variant in *ACSL5*: a marker promoting opposing effect on cell viability and protein expression

**DOI:** 10.1038/s41431-019-0414-5

**Published:** 2019-05-03

**Authors:** Iván Pérez-Núñez, Mohamad Karaky, María Fedetz, Cristina Barrionuevo, Guillermo Izquierdo, Fuencisla Matesanz, Antonio Alcina

**Affiliations:** 1 Department of Cell Biology and Immunology, Instituto de Parasitología y Biomedicina “López Neyra” (IPBLN), Consejo Superior de Investigaciones Científicas (CSIC), 18016 Granada, Spain; 2 Unidad de Esclerosis Múltiple, Hospital Universitario Virgen Macarena, 41009 Sevilla, Spain

**Keywords:** Gene expression, Predictive markers, Genetics research

## Abstract

Long-chain Acyl-CoA synthetases (ACSLs) activate fatty acids (FAs) by thioesterification with Coenzyme A (CoA), generating FA-CoAs. These products are essential for lipid metabolism and carcinogenesis. In previous study, we identified an intronic variant rs2256368:A>G, whose G allele promotes exon 20 skipping in up to 43% of ACSL5 transcripts but its functional relevance is unclear. Here, we compared the expression of splice (Spl) and nonsplice (NSpl) ACSL5 variants and the effect on cell viability under culture conditions that force cells to metabolize fatty acids. We found that lymphoblastoid cell lines from 1000 Genomes Project, bearing Spl genotypes, showed a reduced expression of total ACSL5 protein due to an inefficient translation of the Spl RNA. These cells impaired growth in cultures with phorbol myristate acetate-ionomycin (PMA-Io) or medium deprived of glucose, while production of reactive oxygen species increased in PMA-Io. Specific ACSL5-isoform transfection in HEK239T (kidney), U87 (astroglioma), and HOG (oligodendrocyte) cells showed the Spl protein to be the causal factor of cell-growth inhibition, despite its reduced protein expression. Our findings indicate that the variant rs2256368:A>G can predict a growth inhibitory activity, caused by the Spl isoform of ACSL5 protein, opposed to the activity of the NSpl. Deep understanding of its functioning might have application in metabolic diseases and cancer.

## Introduction

Long-chain acyl-CoA synthetases (ACSLs) activate fatty acids (FAs: C12-C20), by thioesterification with Coenzyme A (CoA), generating FA-CoAs. These products are the initial step in fatty acid metabolism. Once activated, they are involved in cell growth, differentiation, and energy regulation [1–5]. Fatty acids physiologically regulate ACSL expression, but cancer cells could hijack certain involved regulatory mechanisms to deregulate ACSLs. This deregulation is also associated with poor survival in patients with cancer [6].

ACSL5 is implicated in several types of cancers and has a potential prognostic value [6–12]. Unlike other ACSLs, ACSL5 is downregulated in colorectal carcinomas [13, 14], breast [10, 15], bladder [15], and pancreas [12]. However, ACSL5 is overexpressed in glioma [16] and considered a potential therapeutic target [17–19]. In addition, the fibroblast growth factor receptor 2 (FGFR2) ACSL5 chimera RNA rendered clinical gastric cancer cells resistant to treatment with FGFR inhibitors [6, 20].

Excess fatty acid metabolism has increasingly been found to be associated with metabolic disorders and carcinogenesis [6]. Genetic variants in ACSL5 may provide a linkage between the higher prevalence of cancer in obesity. For instance, ACSL5 rs2419621 T allele carriers are more responsive to lifestyle interventions partly due to an increase in the short ACSL5 protein isoform, increasing cellular, tissue, and whole-body fatty acid utilization [21]. Also, ACSL5 rs7903146 is one of the most strongly associated type 2 diabetes loci and resides within a regulatory region of an ACSL5 promoter [22].

The G allele of the rs2256368:A>G variant, located at intron 20 of *ACSL5* gene was identify as the cause of exon 20 skipping in up to 43% of transcript molecules, using expression quantitative trait loci strategy [23]. The present work characterizes the functional effects promoted by this splice (Spl) variant. With this end, we compared the expression of Spl and nonsplice (NSpl) ACSL5 isoforms and the effect on cell viability in lymphoblastoid cell lines (LCLs) from 1000 Genomes Project and in  other cell lines from different  tissues.

## Materials and methods

### Nomenclature and database submission

Rs2256368:A>G variant was submitted to the LOVD 3.0/shared with the submission ID #60251 (http://databases.lovd.nl/shared/view/ACSL5). This variant (hg19 chr10:g.114186624G>A) is located in the *ACSL5* gene (NM_016234.3; c.2079+7G>A). The G allele promotes exon 20 skipping in ACSL5 transcripts (r.2008_2079del) producing spliced (Spl) ACSL5-Δ20 RNAs [23].

### Selection of lymphoblastoid cell lines (LCLs)

Twelve LCLs from HapMap and 1000 Genomes projects, were purchased from Coriell cell repository (Coriell Institute for Medical Research, Camden, NJ, USA). These 12 lines represented the three genotypes of rs2256368:A>G variant: genotype GG in cell lines HG00134, HG00326, HG01048, HG01383; genotype AG in lines GM12004, GM12044, GM12144, GM12717; and genotype AA in lines NA12006, NA11994, NA12043, NA11993. All cells were cultured in RPMI+10% FCS as previously described [24].

### Relative quantification of RNA in LCLs

ACSL5 RNA concentrations in LCLs were measured by real-time reverse transcription (RT) qPCR, normalizing the results to UBE2D2 RNA levels as reported in an earlier study, using the 2E deltaCt (deltaCt = Ct sample-Ct reference) method [25]. The primer sequences were (key: forward-Fw; reverse-Rv; E, exon; 5′–3′ direction): UBE2D2 Fw- CAATTCCGAAGAGAATCCACAAGGAATTG and Rv- GTGTTCCAACAGGACCTGCTGAACAC; non-spliced (NSpl) E20 ACSL5 (using a bridge E19-E20 to E21) Fw-CCAAGTTGTAAGGGAAGCCA and Rv-GCTGTCAATTTGGGTCCGAA; Spl E20 ACSL5 (using bridge E19-E21 to E21) Fw-ACTGTGCCAAAACCAAGTCA and Rv- TGTGCTCATACAGGCTGTCA.

### Cell extracts

Total cell extracts were acquired using RIPA buffer (50 mMTris-HCl pH 7.4, 150 mMNaCl, 1 mM EDTA, 0.5% Na-deoxycholates, 0.1% SDS) plus antiproteases from the Halt Protease Inhibitor Single-Use Cocktail (Pierce, Rockford, IL, USA). Mitochondrial extracts were isolated from LCL NA12006E representing genotype AA, GM12004D representing genotype AG, and HG00134 representing genotype GG, using the Mitochondria Isolation Kit for mammalian cells (Pierce) following the manufacturer’s instructions and using RIPA buffer for protein extraction.

### Western blots

Cell extracts in RIPA buffer were processed for protein separation by sodium-dodecyl sulfate-7% polyacrylamide gel electrophoresis (SDS-PAGE) under reducing conditions and transferred to Immobilon-P transfer membranes (Merck-Millipore Ltd., Cork, Ireland). Blots were incubated with different antibodies from Abcam (Abcam plc, Cambridge, UK). As primary antibodies we used anti-ACSL5 (ab57210) and anti-V5 tag (ab27671); as a secondary antibody, a horseradish peroxidase (HRP)-labeled anti-mouse IgG (ab97023) was used. As a loading control, we used an anti-beta Actin-HRP- labeled antibody (Ab8226) following conditions indicated by the manufactures. Protein bands were detected by an enhanced chemiluminescent substrate for detection of HRP using Pierce ECL Western Blotting Substrate (ThermoScientific, Rockford, IL, USA).

### ACSL5 protein quantification in LCLs

Relative expression of ACSL5 protein was determined by band densitometry of the signal generated on a western blot film, using Bio-Rad’s Image Lab Software (Bio-Rad Laboratories, Hercules, California, USA) as indicated in Supplementary Methods. The band densitometry results were normalized twice: (1) against beta-actin; and (2) against the highest concentration of ACSL5 protein.

### Cell viability of LCLs

The 12 LCLs were cultured at 2500 cells per well in 96-well microtiter plates in three different culture conditions: (i) control cultures (CTL) with the usual growing media indicated above; (ii) cultures treated with phorbol myristate acetate-ionomycin (PMA-Io) [50 ng/ml of phorbol 12-myristate 13-acetate (PMA) plus 10 ng/ml Ionomycin (Io) [Sigma Aldrich, Madrid, Spain]); and (iii) glucose-depleted culture media (no glucose) plus 10% FCS. Growth measurements were performed at 6, 24, and 48 h of culture. Cell viability was determined using the Cell-Titer-Glo Luminescent Cell Viability Kit (Promega, Madison, WI, USA). All readings were first normalized with the readings at 6 h (growth index) and then, the relative growth of each LCL in PMA-Io-treated media or in glucose depleted cultures, were normalized to the growth index of the CTL cultures (relative growth index). Each time point shows the mean growth of four LCLs with the same genotype, and the standard  error of mean (+/-SEM). Each experiment was performed five times for each condition in 12 replicates.

### ROS production in LCLs

ROS production was measured in the 12 LCLs cultured in media treated with PMA-Io, seeded at 5000 cells per well in 96-well microplates for 0.5, 2, 4, and 6 h, using the Cellular Reactive Oxygen Species Detection Assay Kit (Abcam, ab113851), following the manufacturer’s instructions. This kit uses the cell permeant reagent 2′,7′-dichlorofluorescin diacetate (DCFDA) to measure ROS activity within the cell. The resulting oxidation of DCFDA by intracellular ROS was detected by fluorescence spectroscopy with an Infiniti^®^Lumi Microplate Reader (Tecan, Mannedorf, Switzerland) with maximum excitation and emission spectra of 495 and 529 nm, respectively.

### Cloning of ACSL5 isoforms

RNA from one LCL bearing rs2256368 GG genotype, which produced about 60% NSpl and 40% Spl ACSL5 transcripts, was reverse-transcribed (RT) [23]. The cDNA was amplified by PCR using the following primers (in bold is the linker for directional cloning): Fw-**CACC**AGCACGTTAGAAAGCCTGAC and Rv-ATCCTGGATGTGCTCATACA, with the Q5 High-Fidelity DNA Polymerase Kit (New England BioLab, Ipswich, MA, USA), in the following PCR conditions: 1 cycle of 94 °C for 3 min; 34 cycles of 94 °C for 30 s; 60 °C for 30 s; 72 °C for 2 min. The PCR products were cloned into the expression plasmid pcDNA6.2/GW/D-TOPO (Invitrogen, Carlsbad, CA, USA). The resulting plasmid inserts were amplified by PCR from E19 of ACSL5 to the V5 tag sequence in the plasmid using primers: ACSL5 exon (E)19 Fw-CTTCCCTCATTTGCAGCCAA and V5 Rv- CCTAACCCTCTCCTCGGTCT. The PCR conditions were: 1 cycle of 94 °C for 3 min, followed by 34 cycles of 94 °C for 20 s, 60 °C for 20 s, plus 72 °C for 1 min. The plasmids DNAs were validated by Sanger sequencing and purified with an EndoFree Plasmid Maxi Kit (QIAGEN GmbH, Hilden, Germany) for transfection experiments.

### Transfection of different cell lines with ACSL5 isoforms

HEK293 (human embryonic kidney [epithelial]), U87MG (human brain [astroglioma]), and HOG (human oligodendrocyte) [26] cell lines were cultured in DMEM medium (low glucose for HEK) supplemented with 15% fetal calf serum and 100 U/ml penicillin/streptomycin (all from Gibco, Invitrogen. Carlsbad, CA, USA). In all 0.5 × 10^6^ cells/well were seeded on a six-well plate, and transfected with the different recombinant plasmids using lipofectamine LTX reagent (Invitrogen) following the manufacturer’s instructions. The expression of ACSL5 mRNA and ACSL5 protein was analyzed at different times post-transfection, contemplating 48 h post transfection as optimal (determined in preliminary experiments).

### Absolute RNA quantification by digital PCR

RNA was purified from transfected HEK293 cells using the RNeasy Plus Mini Kit (Qiagen, Hilden, Germany) and reverse transcribed into cDNA using Superscript III First-Strand Synthesis SuperMix (Invitrogen) [26]. Expression of ACSL5-V5 mRNA levels was subjected to absolute quantification by droplet digital qPCR (ddqPCR) using QX200 ddPCR Eva Green Supermix and QuantaSoft software (Bio-Rad).

The PCR amplification of the NSpl ACSL5-V5 construct was performed using a bridge primer that hybridizes with sequences in exons 19−20 (Fw-ACTGTGCCAAAACCAAGTTG) and with the sequence of the V5 tag (Rv-AGACCGAGGAGAGGGTTAGG). The PCR amplification for the Spl ACSL5-V5 construct (ACSL5Δ20-V5), was performed using a bridge primer that hybridizes with sequences in exons 19−21 (Fw-ACTGTGCCAAAACCAAGTCA), and with the sequence of the V5 tag (Rv), as indicated above.

### Cell viability of cell lines transfected with ACSL5 isoforms

HEK293, U87MG, and HOG cell lines, transfected with the ACSL5 isoforms, were seeded at 2,500 cells per well in 96-well microtiter plates, at 48 h post transfection. Cell viability was determined using the Cell-Titer-Glo Luminescent Cell Viability Kit (Promega, Madison, WI, USA) following manufacturer’s instructions. Luminescence was measured using Infinite^®^Lumi Microplate Reader (Tecan). The readings at 24 are indicated versus the readings at 6 h. Each experiment was performed three times in 12 replicates.

### Statistical analysis

Statistical analysis was performed with RStudio software. Values are shown as mean ±  SEM  and the number of independent experiments (*n*) performed are indicated above. Data were analyzed by Pearson’s correlation or ANOVA and Bonferroni post-hoc test. A value of *p* < 0.05 was set as the limit of statistical significance.

## Results

### ACSL5 isoforms

**Fig. 1:**
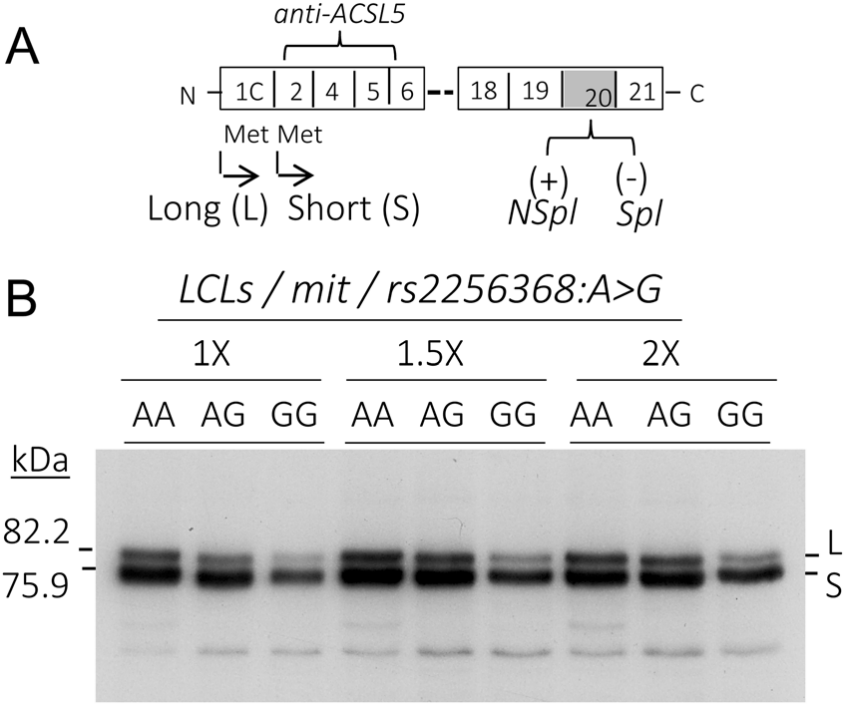
Expression of ACSL5 protein isoforms in LCLs. **a** ACSL5 scheme representing the major protein isoforms. **b** Western blots of mitochondrial (mit) extracts from LCLs bearing the three rs2256368:A>G genotypes (AA, AG, GG) loaded at different concentrations (1X, 1.5X and 2X). The three LCLs expressed different proportions of Spl RNA respect to the NSpl one (AA— 1.8%, AG—14%, and GG—43%). Molecular weights of ACSL5 L and S were calculated from the amino acid sequence

ACSL5 expresses three major transcript isoforms, though only two full-length proteins have been usually observed [1]. Two of them have two in-frame AUG-translational initiators that produce isoforms of different length (Fig. 1a). The longer product (L) is initiated at the first AUG codon, AUG1, corresponding with variant 1. A shorter product (S) is initiated at the downstream AUG2 (v3). A third variant is generated by alternative splicing of the penultimate exon of ACSL5, corresponding with variant v2 (Spl). This Spl isoform is promoted by the genetic variant rs2256368:A>G [23].

### ACSL5 isoforms expressed in LCLs

In order to check the expression of major ACSL5 protein isoforms in LCLs, a western blot of extracts from cells expressing representative % of Spl and NSpl RNAs, with an anti-ACSL5 antibody, was performed (Fig. 1b). Results showed similar band profiles in the three genotypes. However, the Spl-protein bands, with 3.7 kDa less than the L and S isoforms, were not observed. GG-bearing cells, which expressed 40% of SplRNA, showed an important decrease in total ACSL5 protein.

### Expression analysis of RNA and protein isoforms versus genotypes

**Fig. 2:**
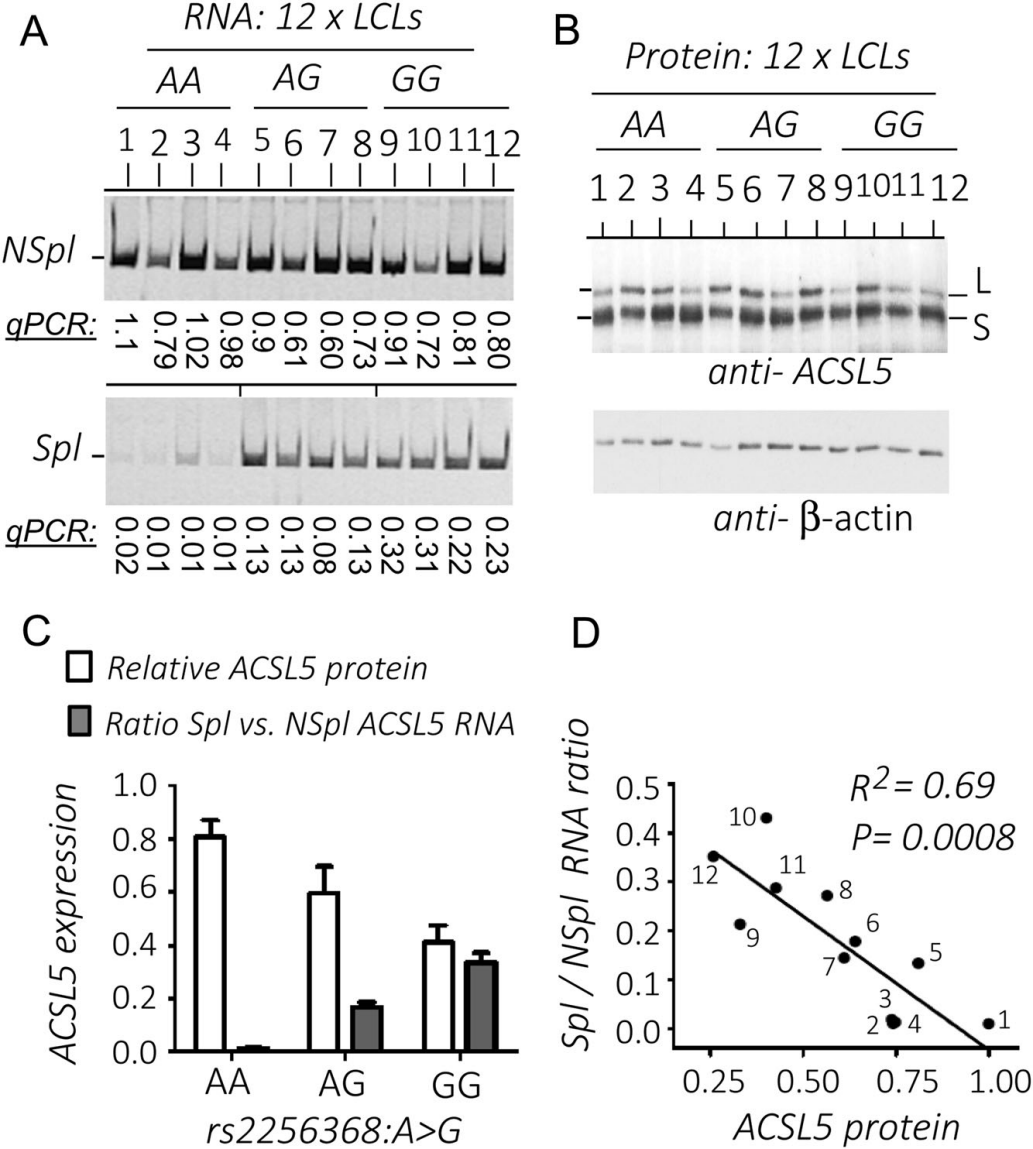
Analysis of ACSL5 expression in LCLs. **a** Quantification of RNA isoforms in 12 LCLs. This panel has been previously published by Matesanz et al. [23], licensed under a Creative Commons Attribution-NonCommercial-ShareAlike 4.0 International License, http://creativecommons.org/licenses/by-nc-sa/4.0/. The panels show end-point PCR amplification visualized in polyacrylamide gel electrophoresis and, additionally, quantified by relative real-time qPCR:(top) Nonspliced ACSL5 transcripts (NSpl); (bottom) spliced ACSL5 transcripts (Spl). **b** Western blots with an anti-ACSL5 antibody of the same LCLs as in the RNA panel. An anti-beta-actin HRP-labeled antibody was used as a loading control for relative protein quantification (“Materials and methods” and Supplementary Material S1). **c** Plot representing relative expression levels of ACSL5 protein (L+S) (white bars) and ratio of Spl versus NSpl RNAs (dark bars). **d** Correlation plot of Spl to NSpl RNA ratios versus relative ACSL5 protein levels. Pearson correlation was used to estimate determination coefficient (*R*^2^) and *P*-value

For the purpose of finding an explanation to the previous observations, we carried out an expression analysis of RNA and protein isoform levels in 12 LCLs, four of each genotype (Fig. 2). For instance, the LCL no. 10, carrier of GG genotype, produced 43% of Spl RNA (Fig. 2a), but the Spl protein was not apparently detected (Fig. 2b). The correlation analysis showed that the increase of Spl RNA, and the G-allele doses, was linked to the decrease of total ACSL5 protein (Fig. 2c). The high determination coefficient (*r*^2^ = 0.69) suggested that the major part of the ACSL5 protein reduction corresponded to the increase of Spl RNA (Fig. 2d). All of these data suggested that the expression of Spl-ACSL5 protein was downregulated as compared to the NSpl isoforms.

### Ectopic expression of Spl and NSpl isoforms

**Fig. 3:**
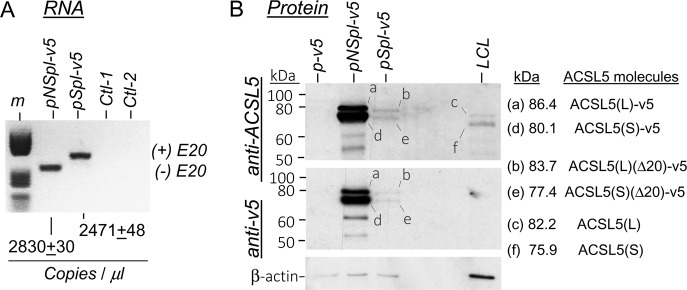
Expression analysis of Spl and NSpl constructs in transfected HEK293 cells. **a** Droplet digital PCR quantification of RNA expression from the spliced (pSpl) and nonspliced (pNSpl) cDNAs, visualized in agarose-gel electrophoresis. Ctl-1 and Ctl-2 were PCR negative controls (RT performed without RT enzyme) for each isoform. Lane m was loaded with a DNA size marker mix (Marker V from Roche). **b** ACSL5 protein expression analysed by western blots of extracts from the transfected cells. Lane LCL is a positive control extract containing endogenous ACSL5 from one LCL, for comparison with the recombinant constructs from transfected cells. Lane kDa represents the position of molecular size markers. Letters and lines inside the blots are explained on the right site, representing the protein sequence-calculated molecular weights of the different ACSL5 molecules detected

To elucidate if the SplRNA was translated into protein, HEK293 cells were transfected with the Spl and NSpl cDNA constructs. The results showed that the both RNA isoforms, Spl and NSpl, were expressed similarly in the corresponding transfected cells (Fig. 3a). However, a dramatic decrease of Spl-protein levels was observed, without signs of differential degradation. Thus, these data suggested that the Spl-RNA was translated into protein in a very inefficient way, compared to the NSpl-isoform. In addition, these data explained why the Spl protein, endogenously expressed in LCLs bearing GG genotypes, was not detected.

### Cell viability of LCL bearing Spl and NSpl genotypes

**Fig. 4:**
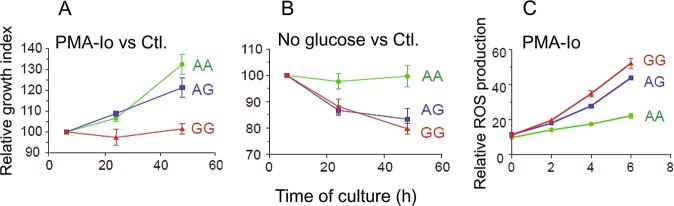
Relative growth index of 12 LCLs, four of each rs2256368:A>G genotype. Cells were cultured under conditions that force cells to use long-chain fatty acids and compared with the growth in control cultures. Student *t*-test was used to estimate difference between each genotype group. *P*-values (*P*) at 48 h of culture were in **a** *P*(AA versus AG) = 0.0487; *P*(AG versus GG) = 0.0013; *P*(AA versus GG)<0.0001. **b** *P*(AA versus AG) = 0.0095; *P*(AG versus GG) = 0.463; *P*(AA versus GG) = 0.0003. **c** Relative ROS production of the 12 LCLs in presence of PMA-Io

Given the implications of ACSL5 in mitochondrial functions, we measured the ATP content (correlating with cell growth) in the 12 LCLs bearing the three rs2256368:A>G genotypes (Fig. 4), under mitochondrial-related stress conditions. Thus, cells were cultured in culture media with PMA-Io, stimulating lymphocyte cell growth [25] (Fig. 4a), which increased the mitochondrial function; or in culture media depleted of glucose, called “No glucose” in the figure (Fig. 4b), forcing cells to use exogenous long-chain fatty acids as energy source. Growth of each treated culture was referred to the growth at 6 h and then to the corresponding untreated culture (relative growth index). Cells bearing AA genotype, stimulated with PMA-Io, responded increasing growth more than the untreated cells and were not affected by absence of glucose within 48 h of culture, compared with standard medium with glucose and without PMA-Io. By contrary, cells bearing AG or GG genotypes were significantly affected, reducing the growth under PMA-Io stimulation, and in cultures depleted of glucose, respect to the control cultures. These findings suggested an association of the impaired cell growth capability with the G allele carriers under mitochondrial stress. Thus, expression of the SpL protein and/or decrease of total ACSL5 protein were the two variables associated with these growth effects.

### Reactive oxygen species

To assess whether the impaired cell growth of LCLs bearing the G allele was related with the oxidative stress response, cells were stimulated with PMA-Io up to 6 h. Treatment with PMA-Io resulted in ROS production, which was always higher in cells expressing the Spl variant (GG bearing cells). The results were normalized to the positive control treatment, using tert-Butyl hydroperoxide (Fig. 4c). All these data suggested that the cells containing the rs2256368 G allele were negatively affected when mitochondrial-related stressors were present.

### Identification of causal isoform of the growth inhibition

**Fig. 5:**
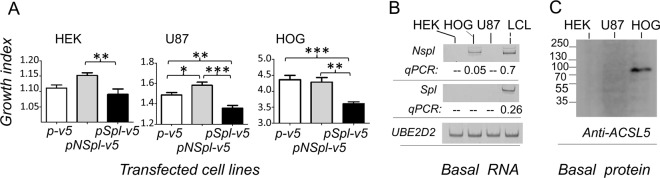
Effect of ACSL5 isoforms on cell viability. **a** Growth index of cells transfected with control plasmid (p-V5), nonspliced (pNSpl-V5) and spliced (pSpl-V5) constructs. Statistical significance is indicated with asterisks: *P < 0.05; **P < 0.005; ***P < 0.0005. **b** Basal level of ACSL5 RNA in nontransfected cells. One LCL is included to show the endogenous expression of Spl and NSpl isoforms, as a positive control. These data were obtained by real time qPCR and the amplified samples were run in a polyacrylamide gel electrophoresis. UBE2D2 expression was used as a reference. **c** Basal level of ACSL5 protein expression performed by western blots of nontransfected cell lines

Finally, to identify the ACSL5 isoform responsible for the growth inhibition, different cell lines (HEK293, U87, and HOG) were transfected with the different ACSL5 constructs (Fig. 5a). Thus, the cells transfected with the Spl construct (pSpl-V5) showed lower growth capability than the control cultures transfected with the plasmid (p-v5), as opposed to the growth capability of cells transfected with the NSpl construct (pNSpl-V5). Therefore, despite its low production, the Spl-V5 protein impaired cell growth in comparison to the NSpl-V5 and p-V5. On the other hand, ROS production (data not shown) was increased in all of these cell lines expressing recombinant Spl-V5 protein, but with no statistical significance, except U87 cells. This could happen because the induction times of ROS and the expression of the cDNA-V5 constructs did not coincide (data not shown). All these cells expressed the ACSL5 constructs in a similar way as shown in transfected HEK293 in Fig. 3b, but HOG cells expressed a higher ratio of Spl-V5 protein to NSpl-V5 (data not shown). Basal expression of RNA and protein was detected neither in HEK293 nor in U87. However, HOG cells expressed a minimal amount of RNA and protein (Fig. 5b, c).

## Discussion

This study aimed at analyzing the functional role of the genetic variant rs2256368:A>G whose G allele promotes exon 20 skipping in up to 43% of ACSL5 transcripts [23]. We found that the Spl RNAs were translated into Spl proteins very inefficiently, as compared with the translation levels of the NSpl isoforms.

The differential production of Spl and NSpl proteins was clearly observed in LCLs bearing GG genotype, expressing both RNA isoforms, as well as in cell lines transfected with the two cDNA constructs. In this last case, the Splprotein expression was drastically reduced compared with the NSpl isoform, from similar number of corresponding mRNA molecules. Consequently, one or several mechanisms of translation regulation, that take place in the cell, might be affecting the production of the Spl protein.

It has been described microRNAs (miRNAs) that block the translation initiation without detectable changes in mRNA levels [27, 28]. For the more highly repressed mRNA translation, its destabilization explains most miRNA-mediated repression [29]. Another possible mechanism could be based on the cell quality control, preventing synthesis of aberrant Spl proteins at the ribosome [30, 31]. Aberrant, misfolded, and mislocalized proteins are often toxic to cells and result in many human diseases. Although this mechanism could also affect the Spl protein, we did not observe evidence of specific differential degradation.

We showed that the Spl protein was responsible of the growth-inhibitory effect, despite its low protein expression. The evidence were obtained from LCLs expressing the Spl isoform, cultured in conditions that forced cells to use long-chain fatty acids, as well as from cell lines transfected with the two ACSL5 isoforms. In both cases, the growth-inhibitory effect caused by the Spl protein was opposed to the growth-promoting activity of the NSpl isoform, inhibiting cell growth below the level of control cells. These results suggested that the activity of the Spl protein had a certain toxic effect, which would be attenuated by inefficient translation of the Spl variant. Using a conceptual explanation from Battle et al. [32], it seems that this “detrimental” impact on cell growth is being attenuated or buffered by a large reduction of protein production [30, 32, 33]. In addition, our data also support that NSpl ACSL5 levels may be essential to reduce ROS-related stress, driving to a correct cell growth.

A detrimental alternative splicing that involves exon 7 skipping in the SP140 transcripts has also been reported [34, 35]. The genetic variant that promotes this alternative splicing is associated with multiple sclerosis, Crohn’s disease, and chronic lymphocytic leukemia. However, what associates with the indicated diseases is the reduction of the NSpl protein expression because the Spl protein variant was not expressed.

Exon 20 skipped variant of ACSL5 protein (Spl) was studied for the first time by Gassler et al. [36]. They revealed that the ratio of the ACSL5-full length and the Spl- ACSL5 protein increases along the crypt-villus axis of human small intestine. This fact was considered of functional relevance for apoptosis of senescent enterocytes at the villus tip and suggested to be involved in the pathogenesis of several intestinal disorders such as intestinal neoplasia [36]. It is unknown if the Spl ACSL5 isoform was promoted or not by the genetic variant analyzed in this study. Given that ACSL5 is highly expressed in human small intestinal mucosa, it would be possible that a small amount of the transcript is Spl even in the nonsplicing genotype AA.

The different allelic carriers of this genetic variant may produce large differences in the expression of the Spl transcripts (expression quantitative trait loci, eQTL) and protein isoforms (protein quantitative trait loci, pQTL), thereby it can increase phenotypic variability and the susceptibility to complex traits in human populations [30, 33, 37, 38]. Future association studies are necessary to find conditions in which this variant is involved.

As a marker, this genetic variant may be useful to predict the presence of an Spl RNA besides of the corresponding Spl protein. Its low expression level seems to attenuate its strong growth-inhibition effect. ACSL5 is generally decreased in those cancer types where ACSL1 and ACSL4 are upregulated [6], so it would be interesting to deepen our understanding of the mechanisms implicated in this alternative splicing, because it seems that between both isoforms there is a potential response against the two type of cancer cells, the ones that upregulate ACSL5 and the ones that downregulate it. All of these results may be of relevance in conditions involving lipid metabolism and carcinogenesis.

## Supplementary information

Supplementary Material S1
