# Supplementary figures and images for: Splice-site variant in ACSL5: a marker promoting opposing effect on cell viability and protein expression
Source: Eur J Hum Genet. 2019 May 3;27(12):1836–44. doi: 10.1038/s41431-019-0414-5 (PMC6871522; doi:10.1038/s41431-019-0414-5)

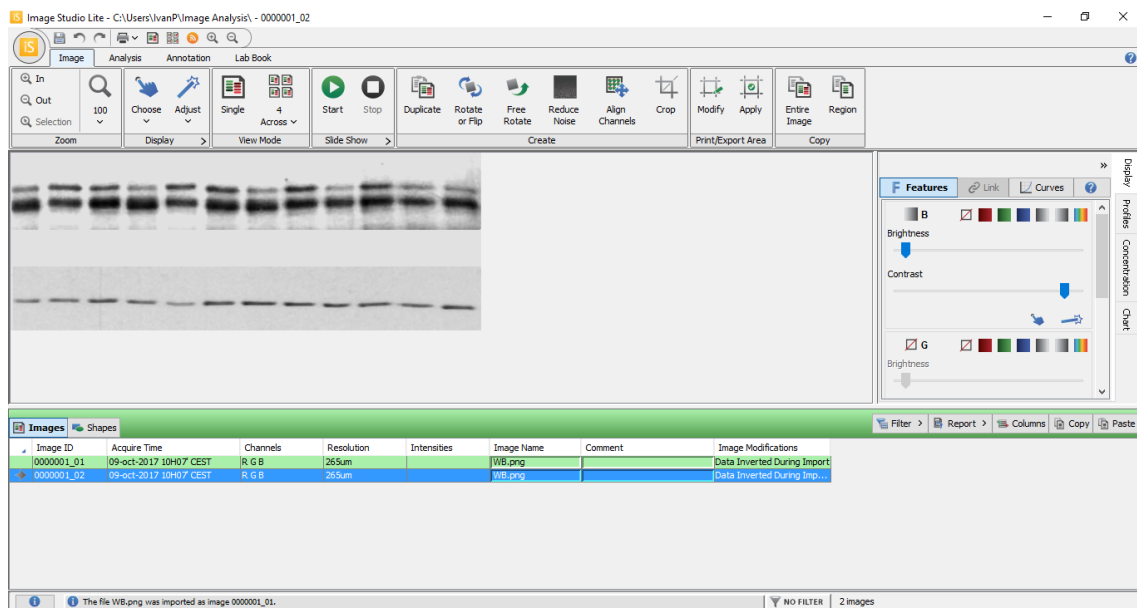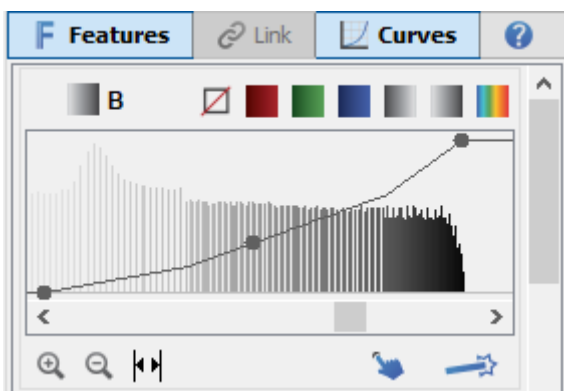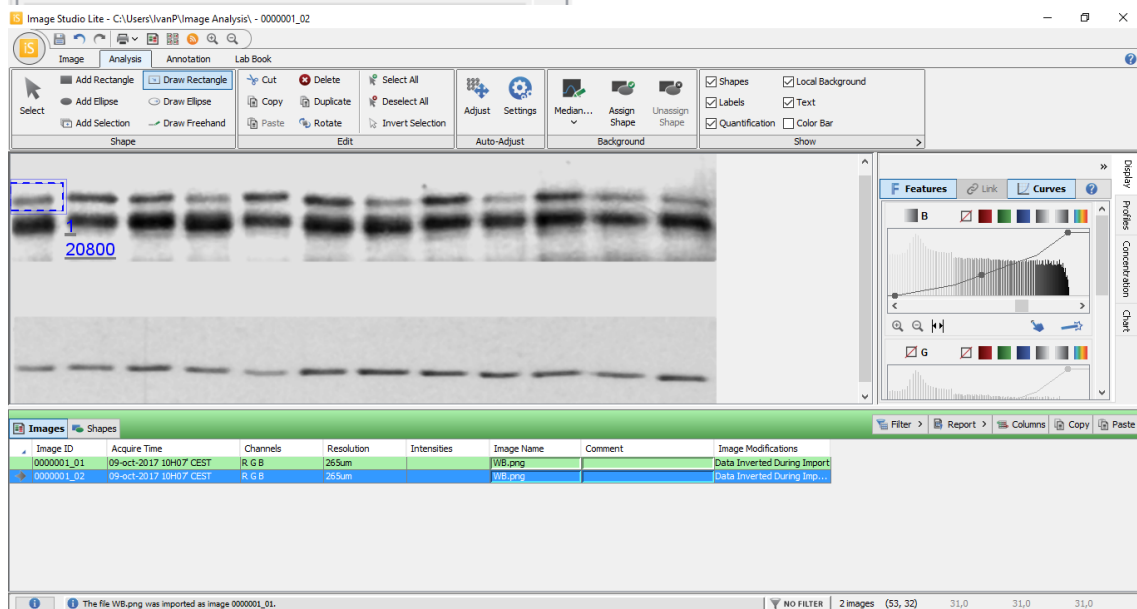

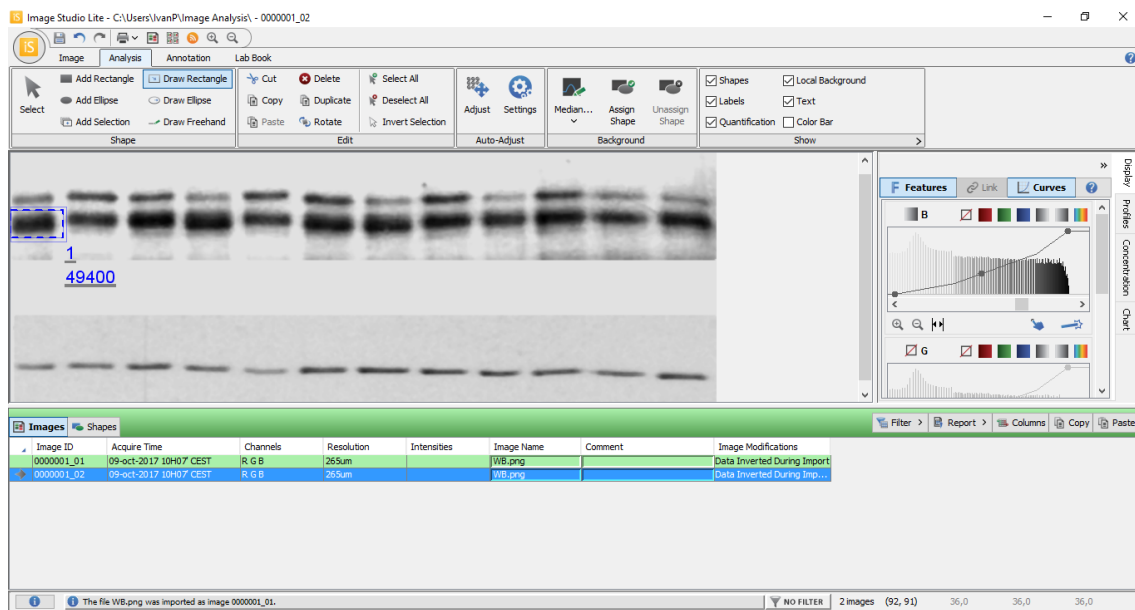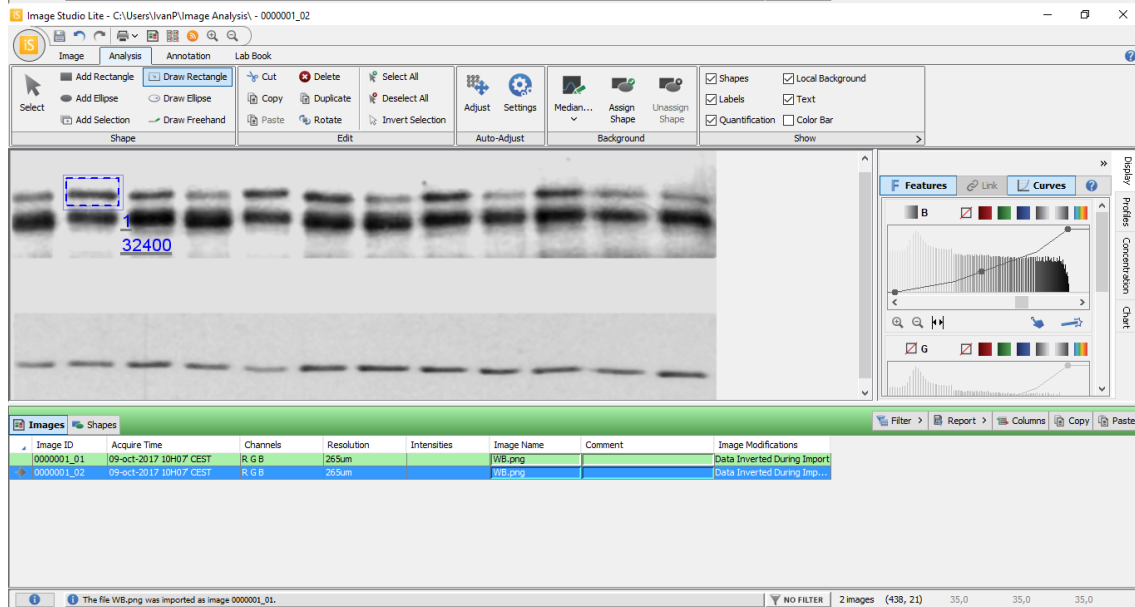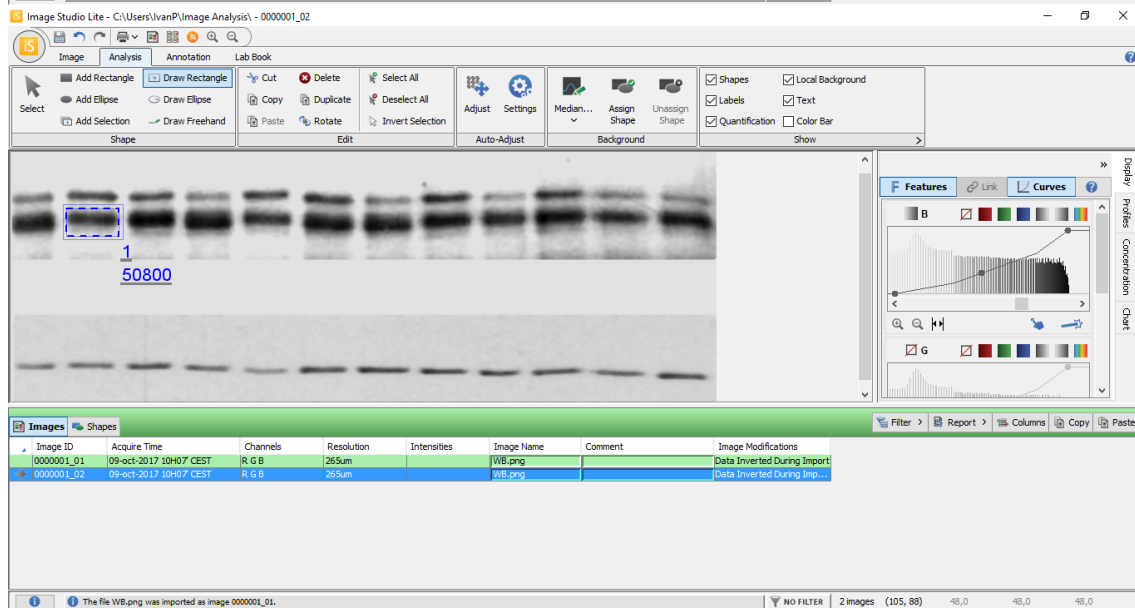

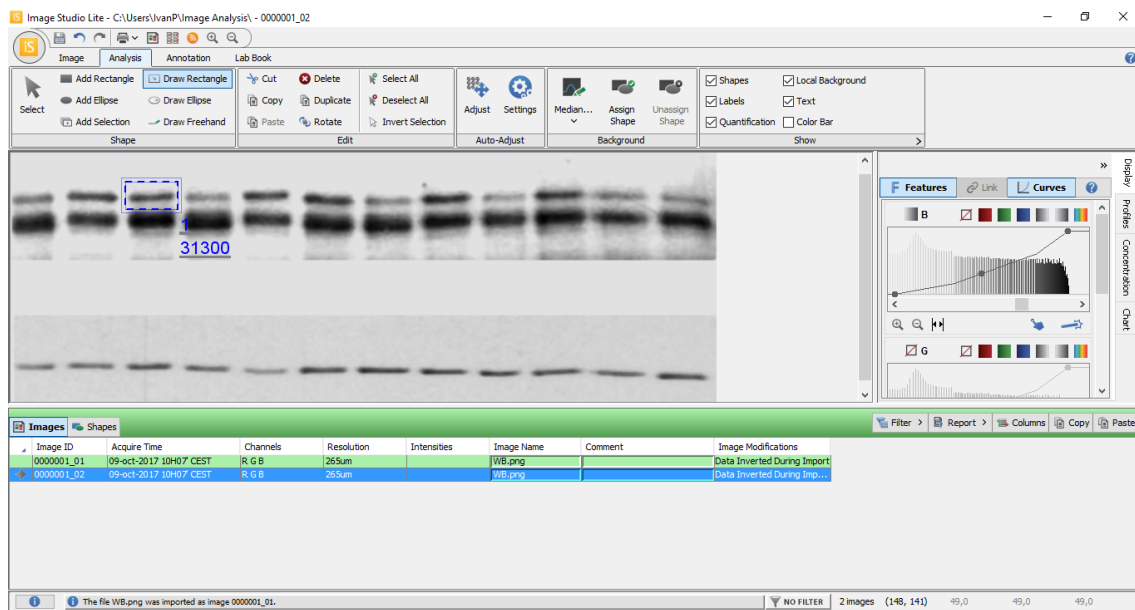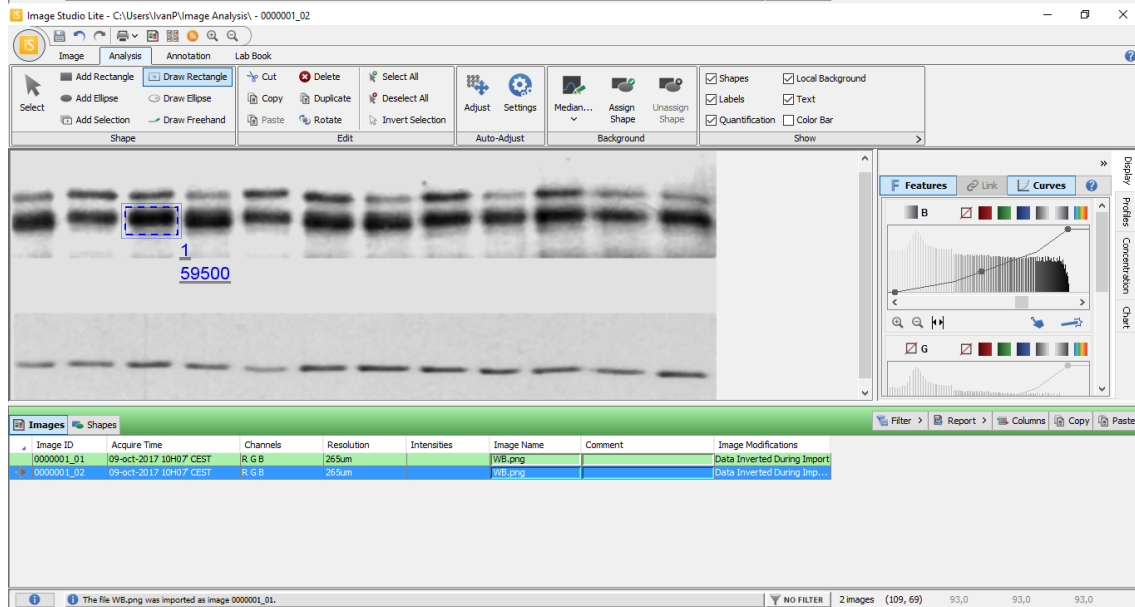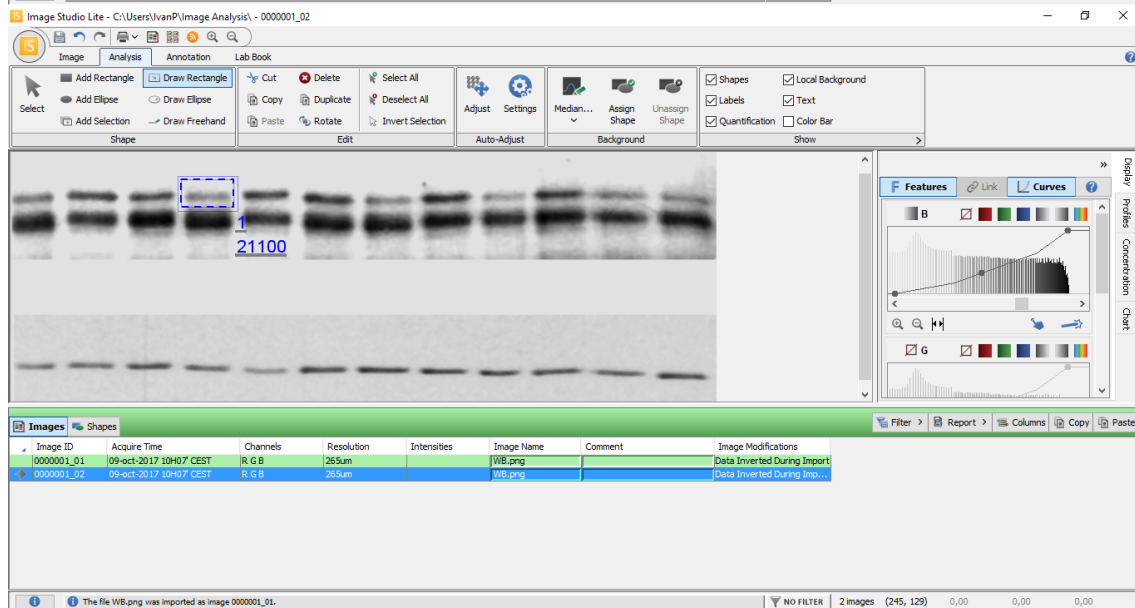

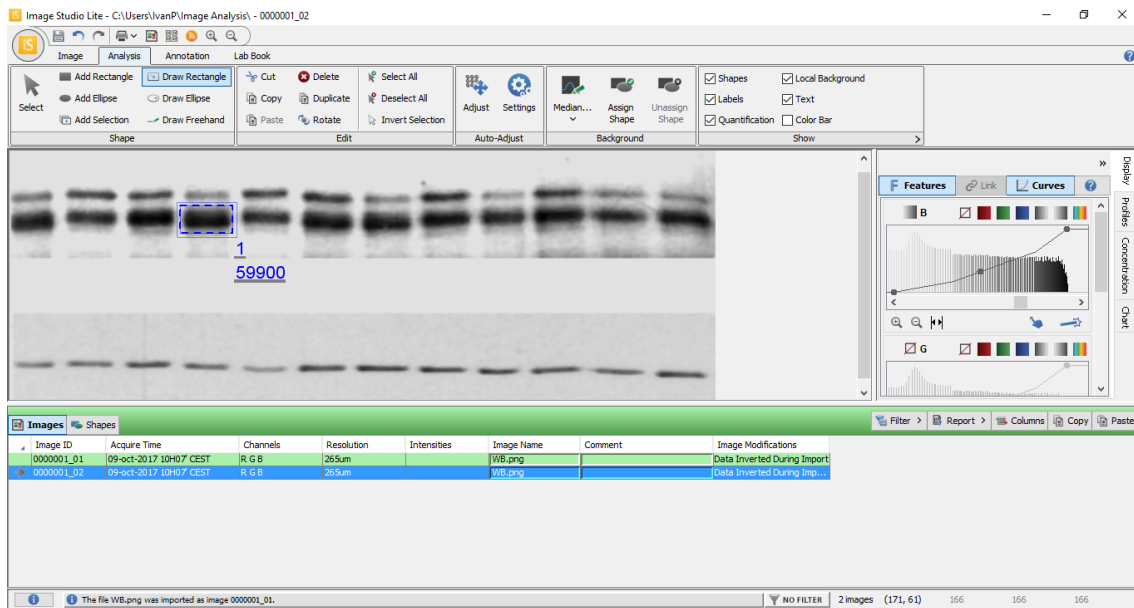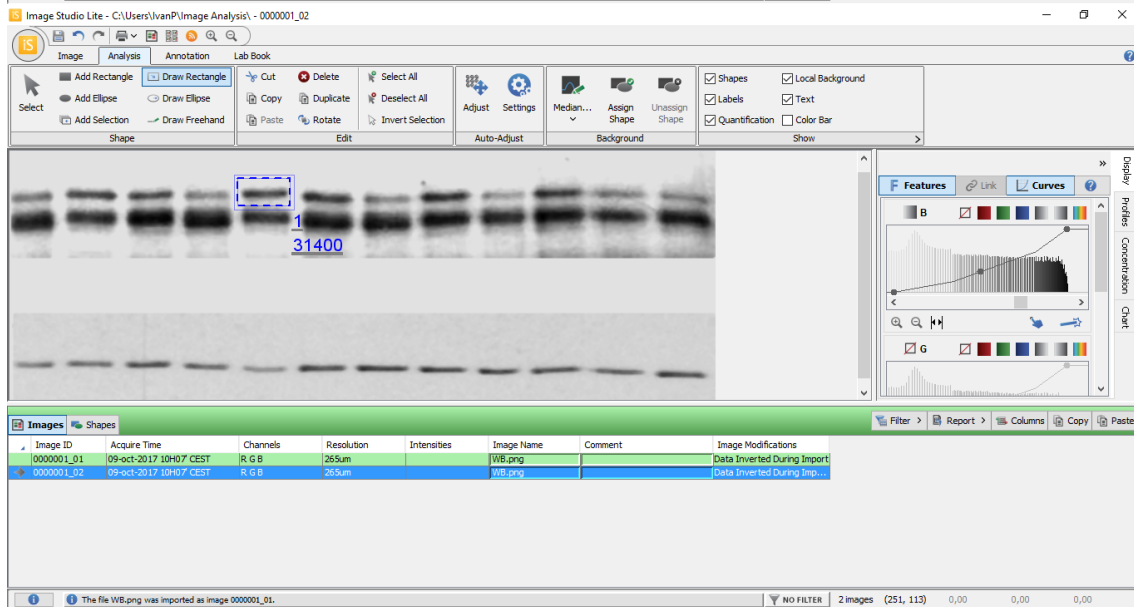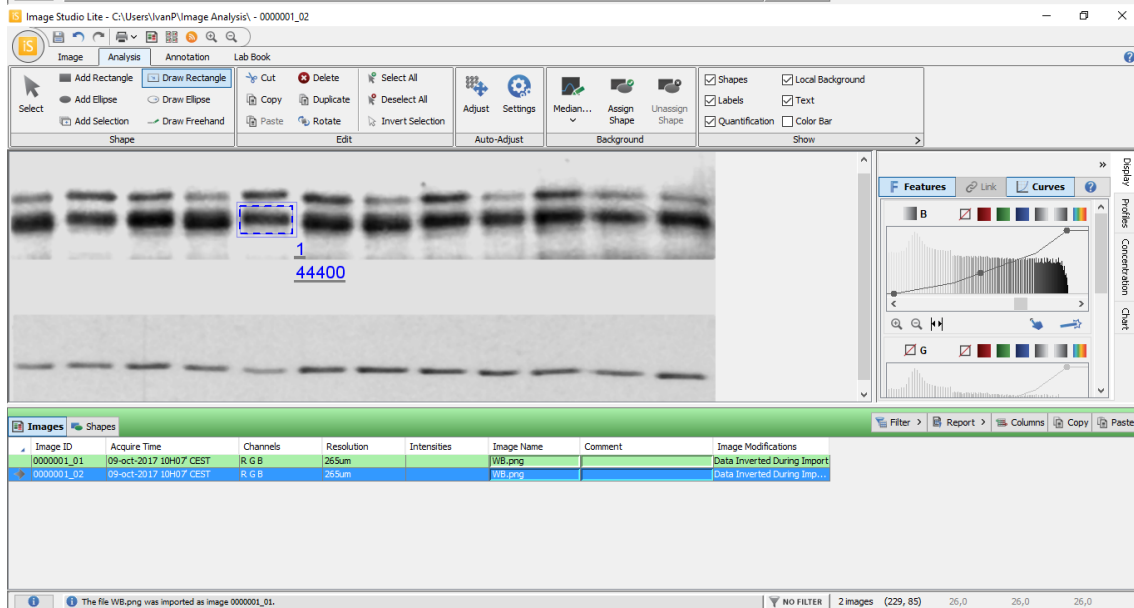

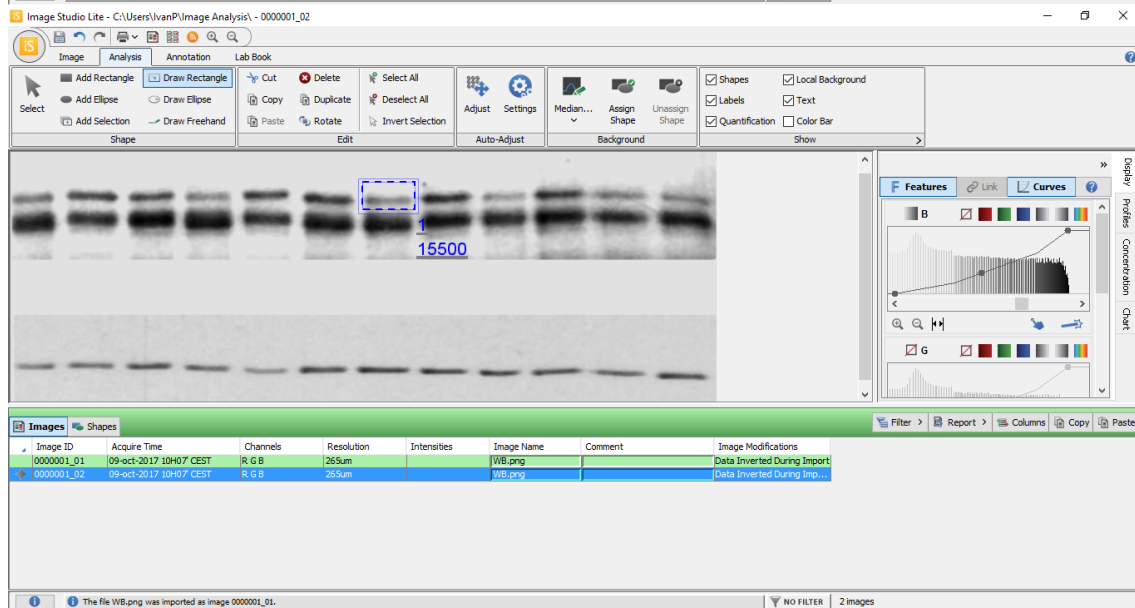

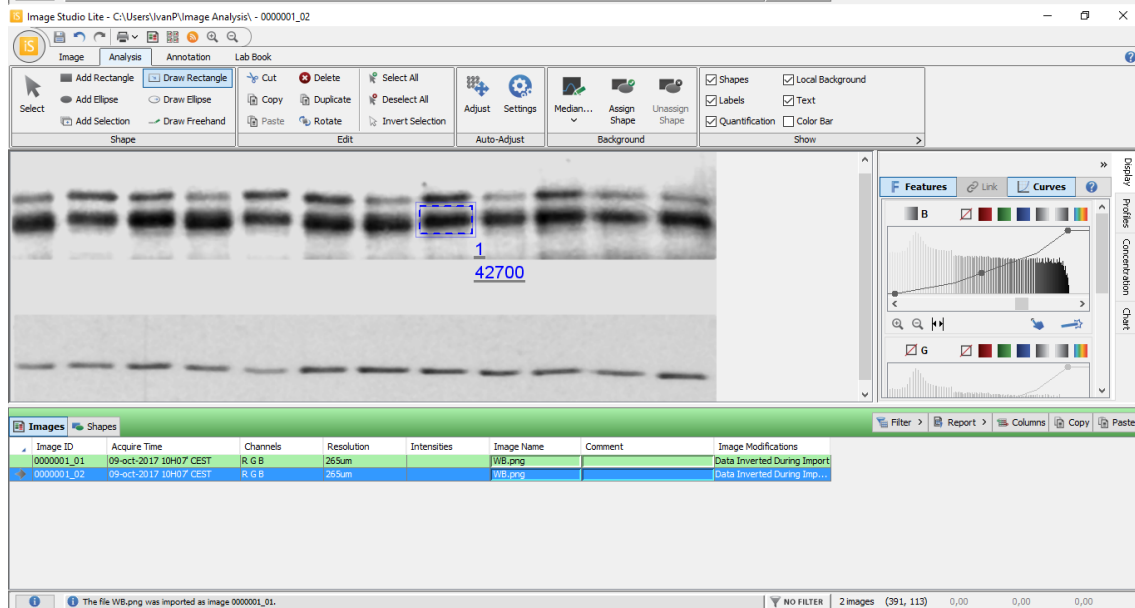

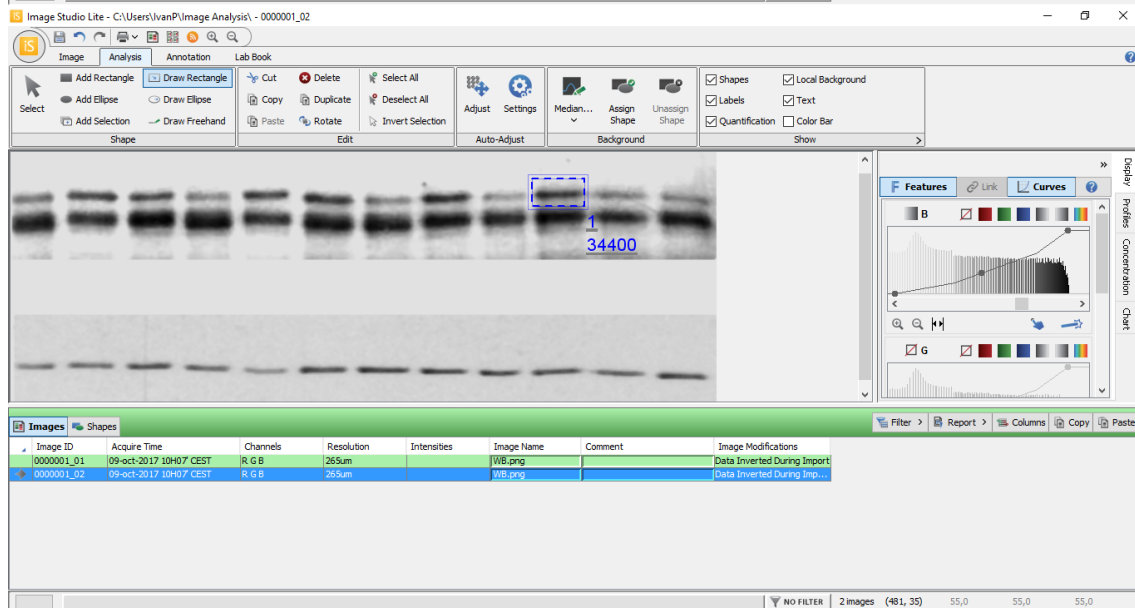

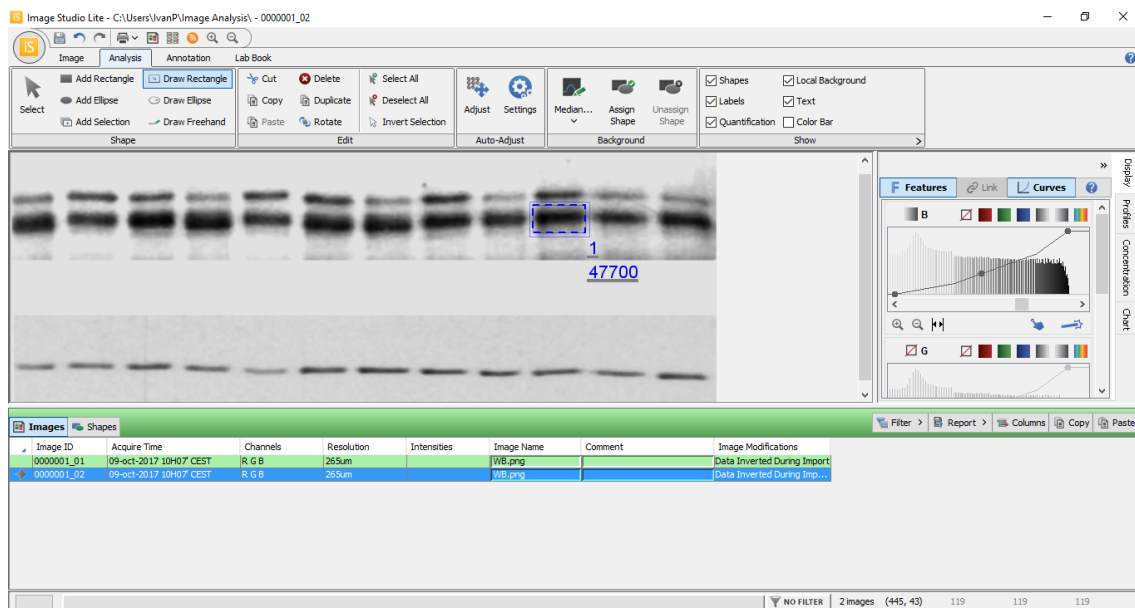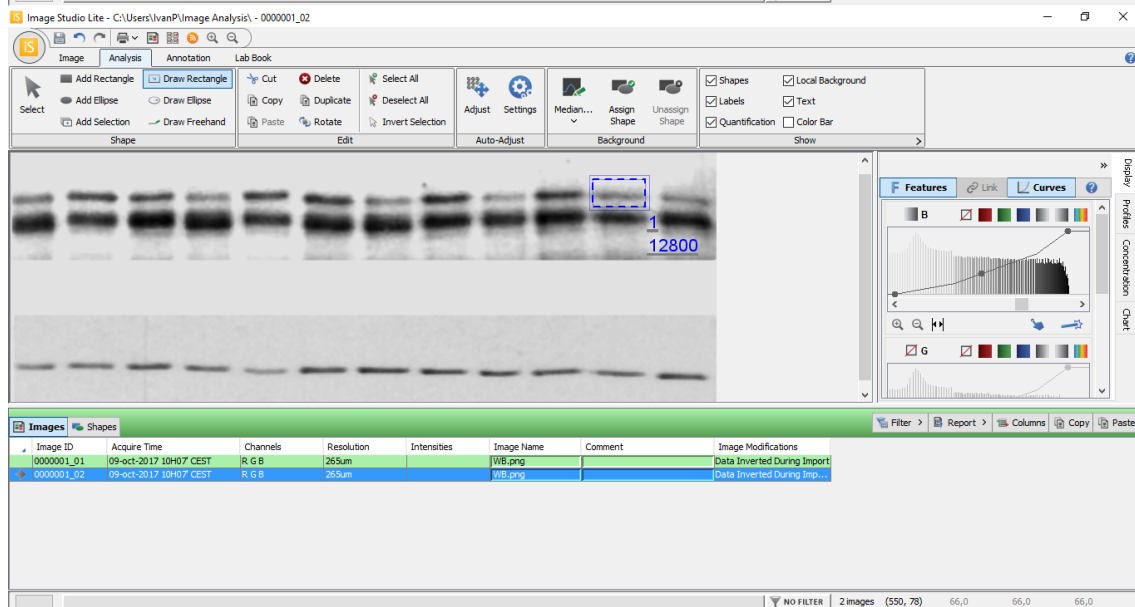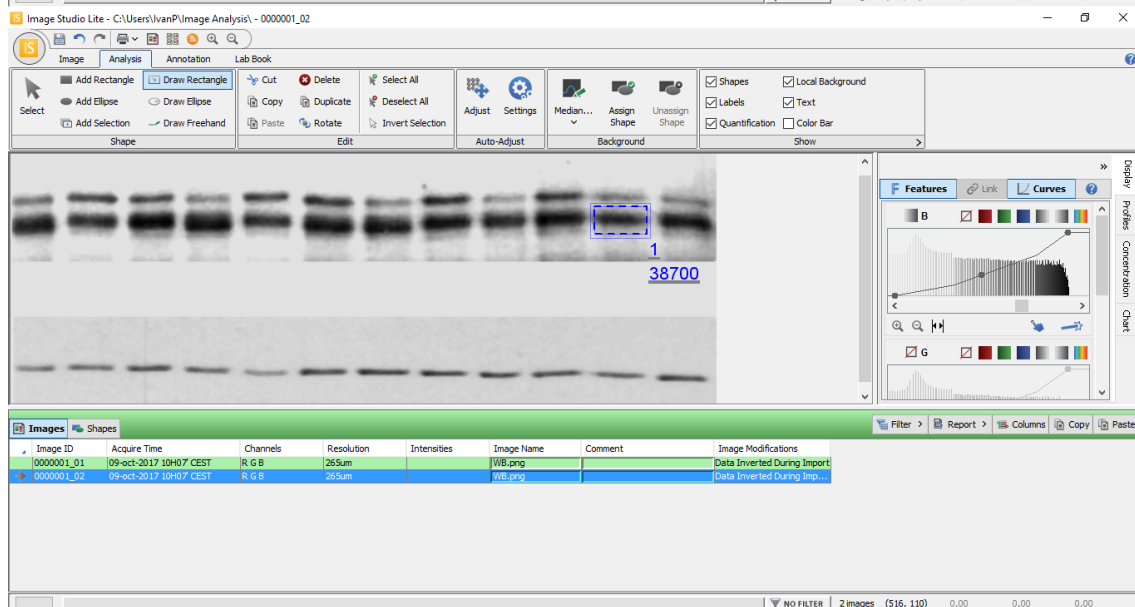

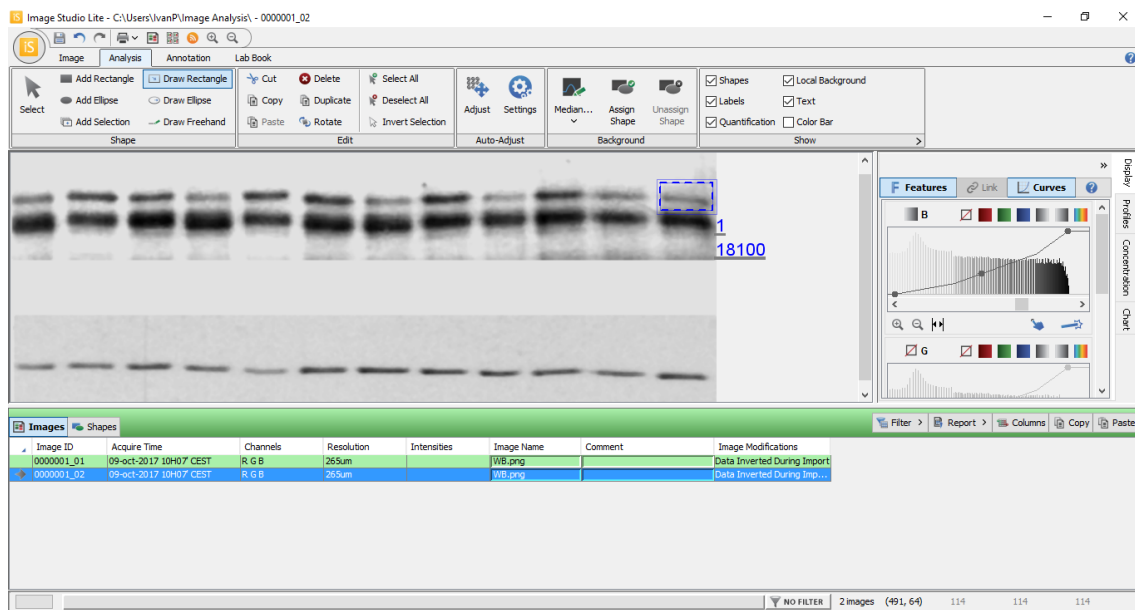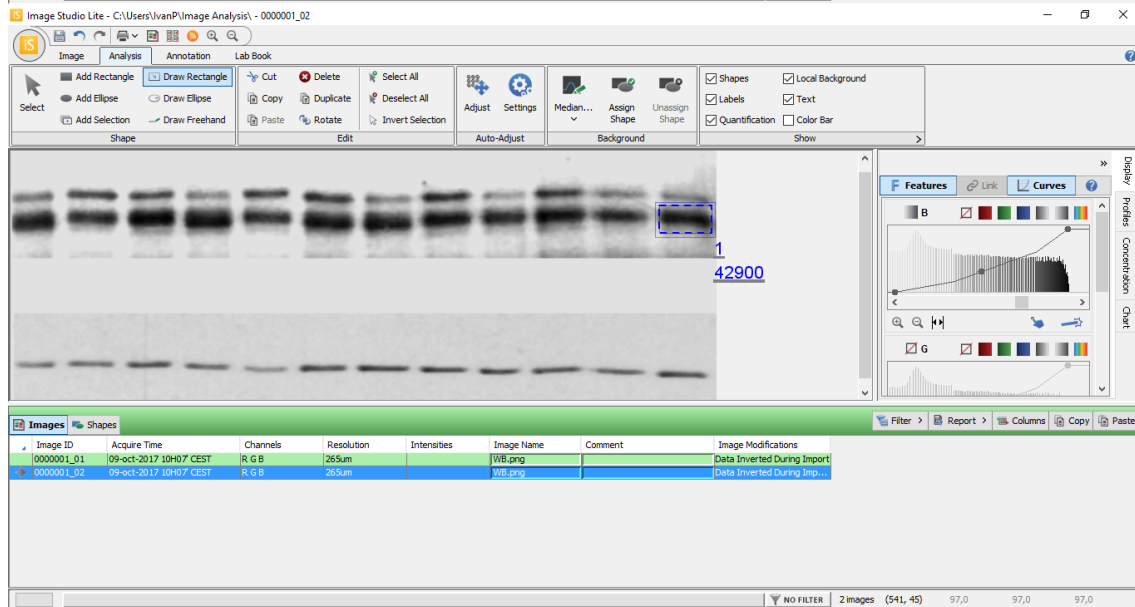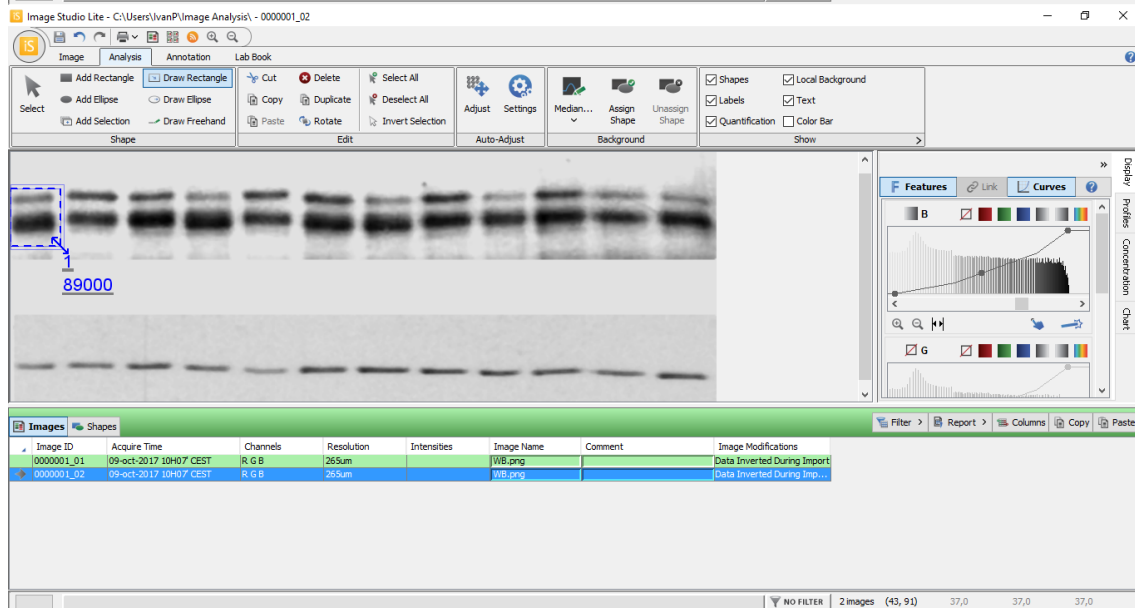

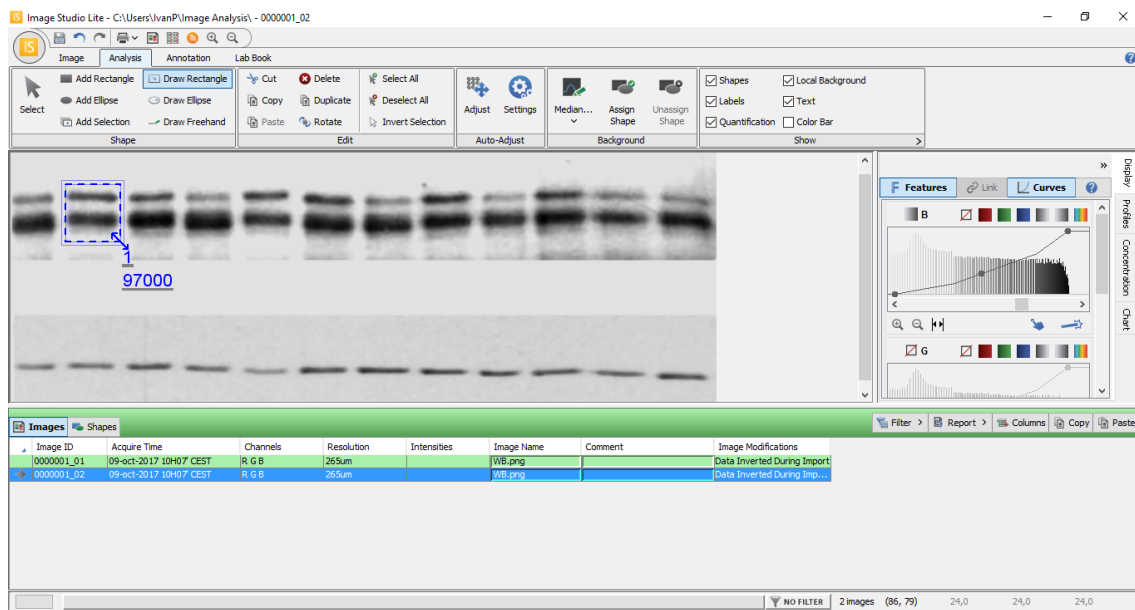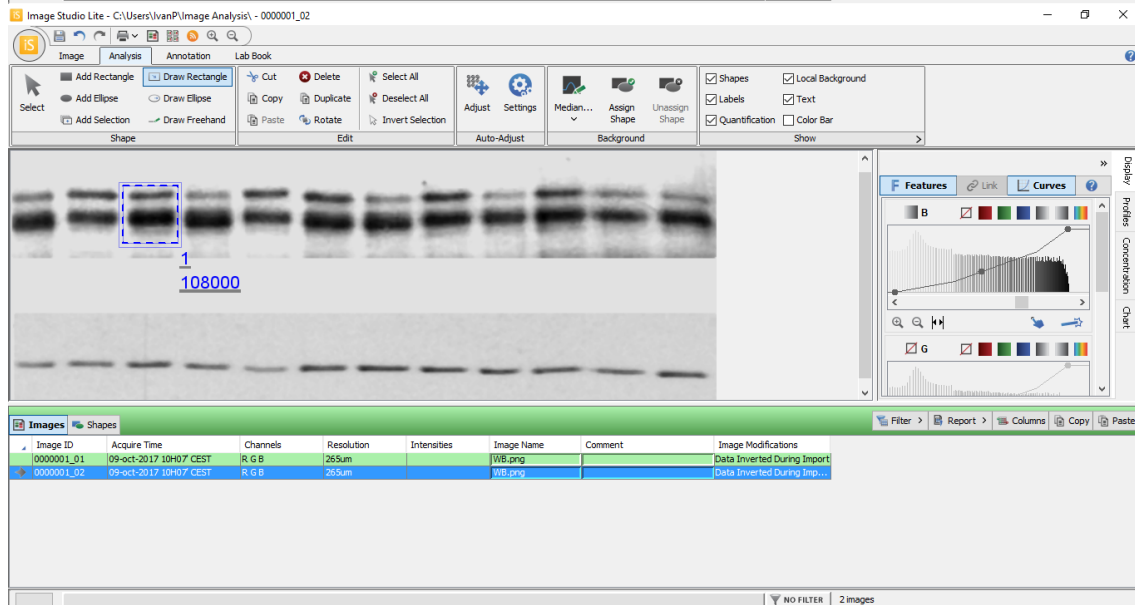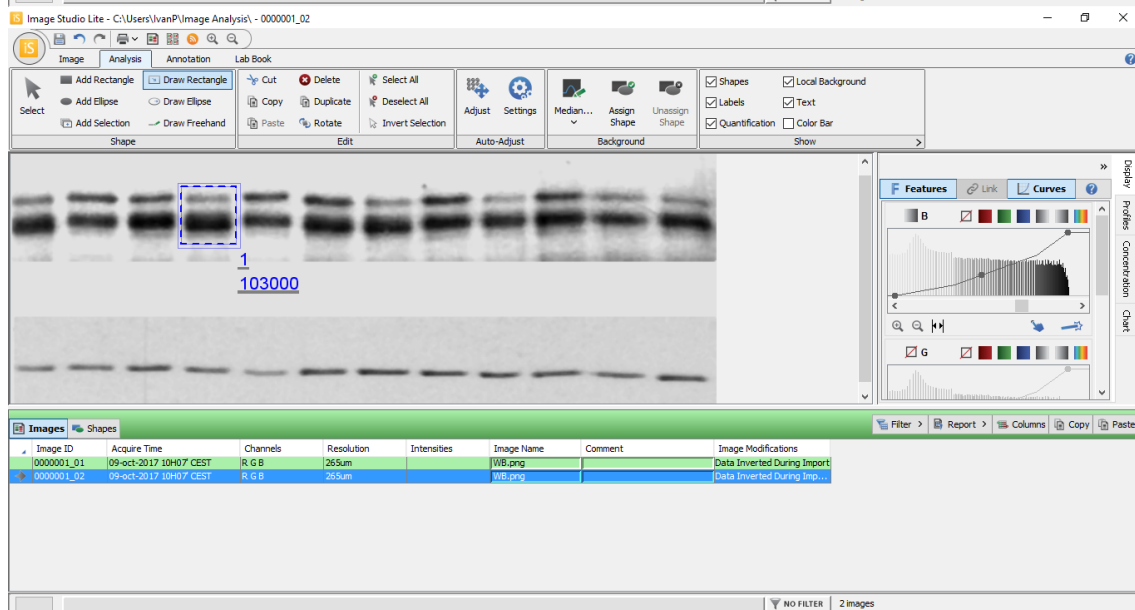

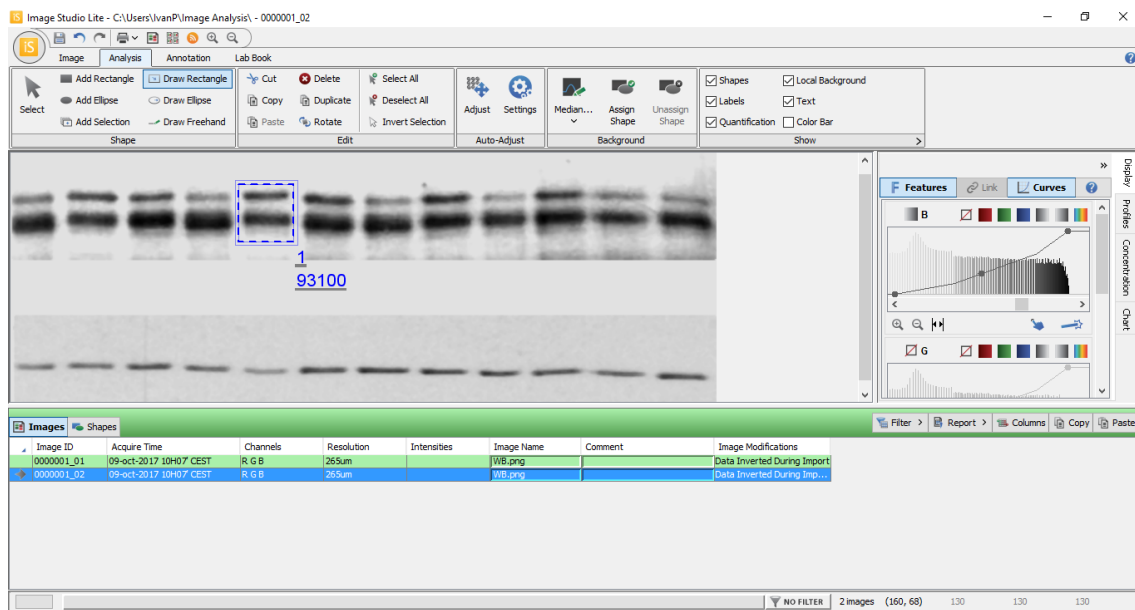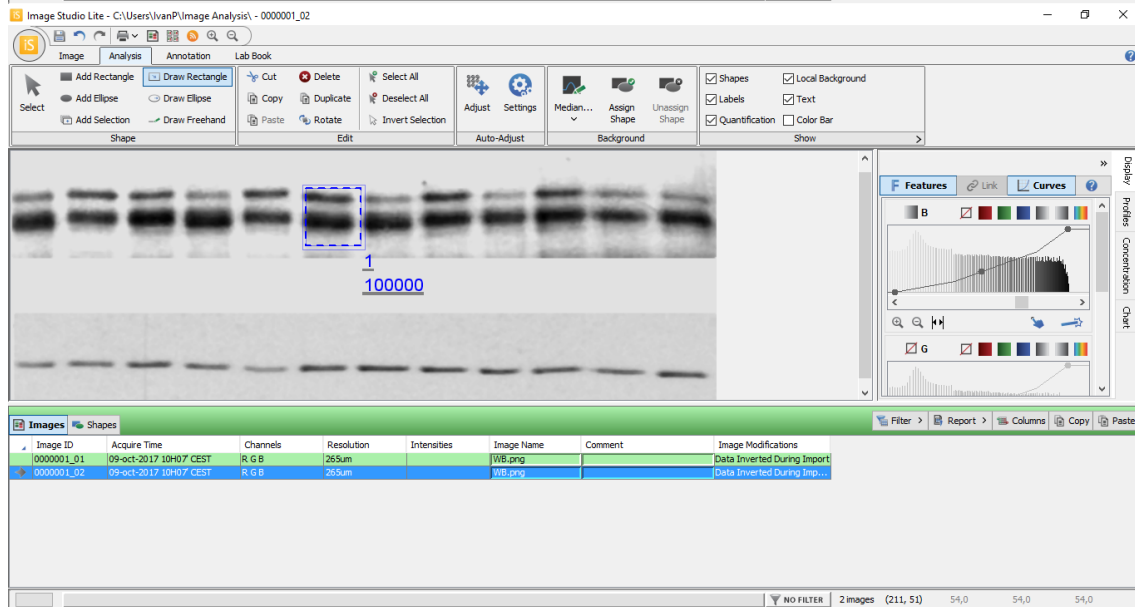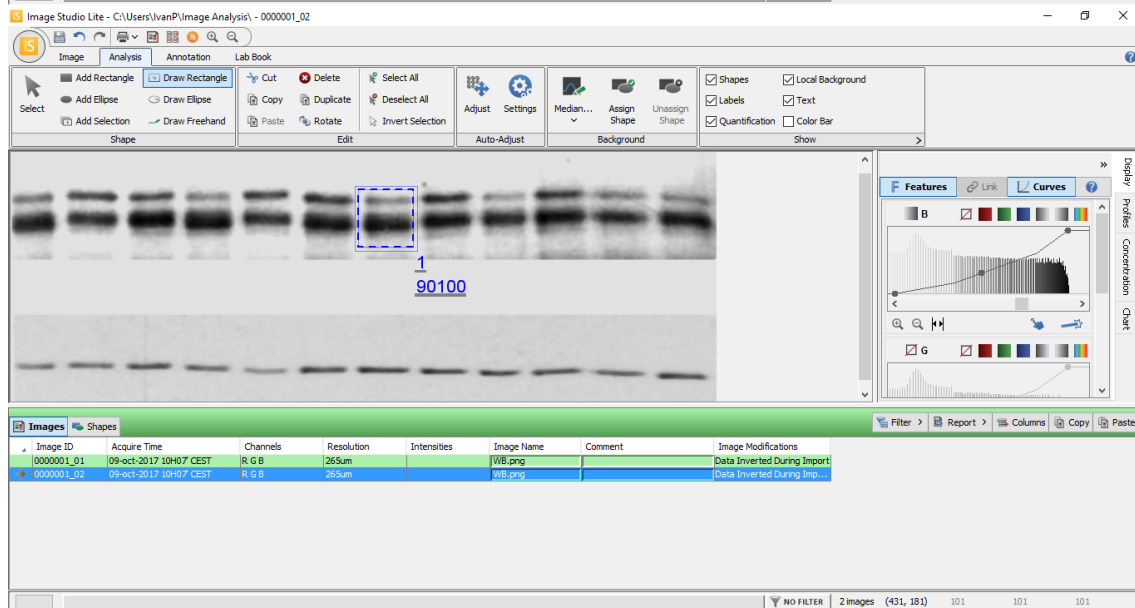

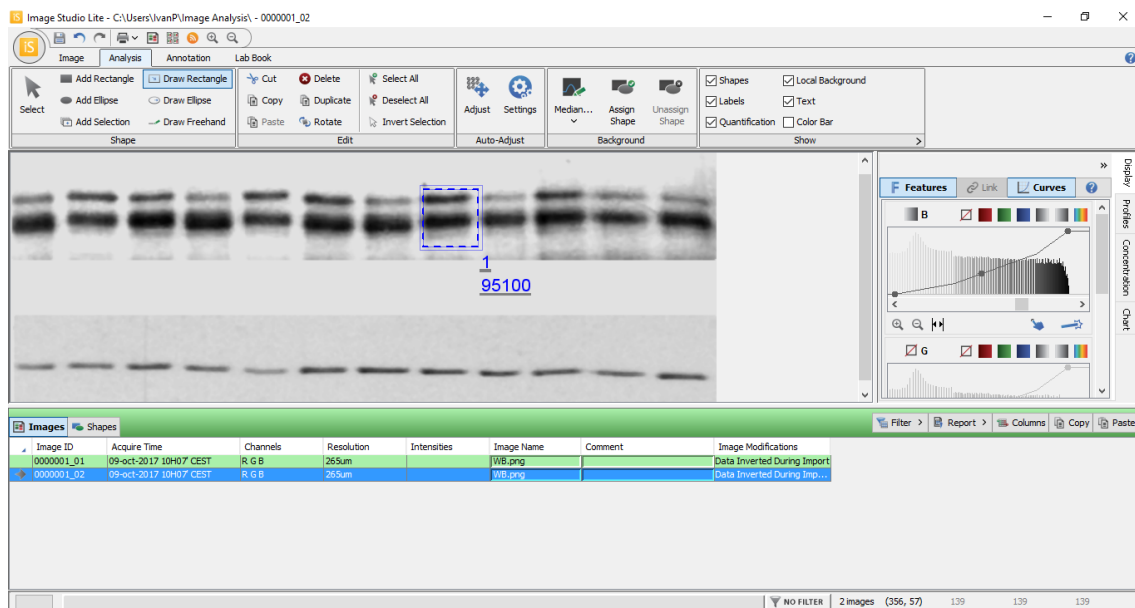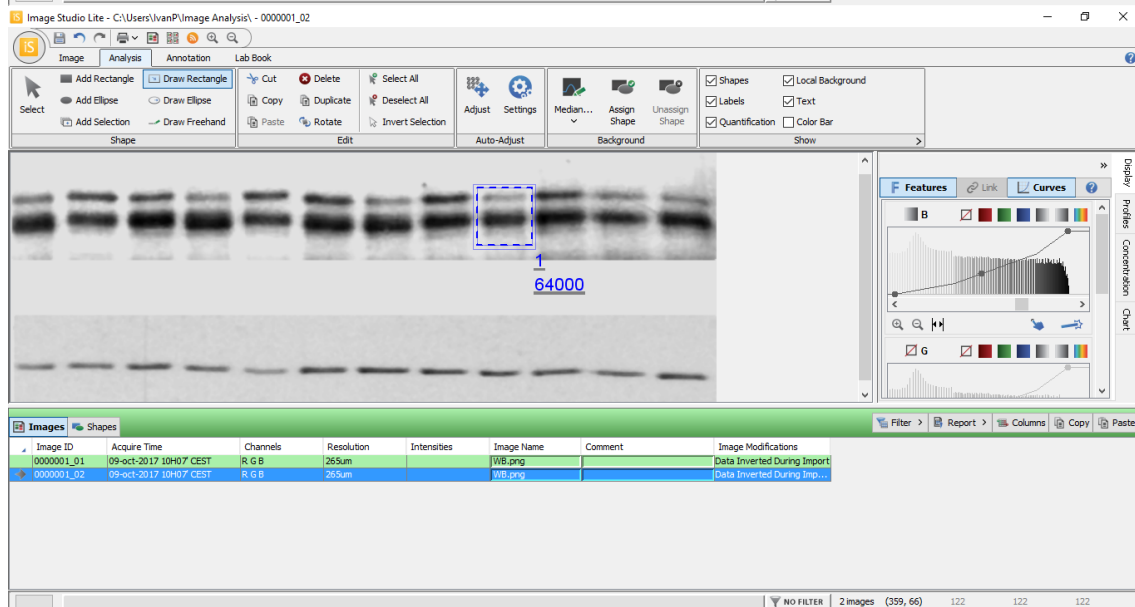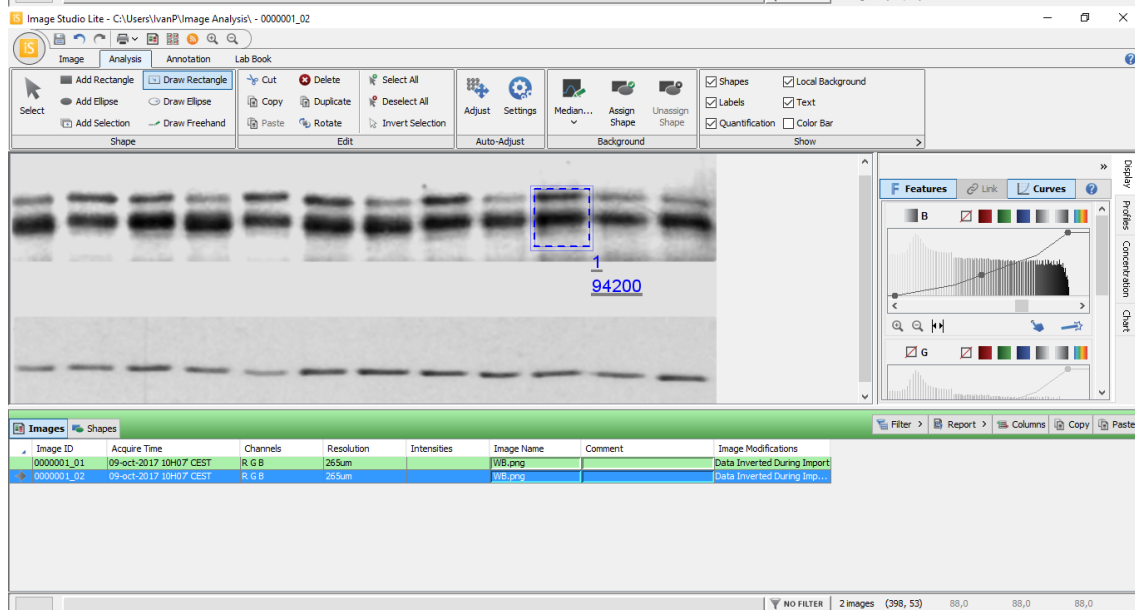

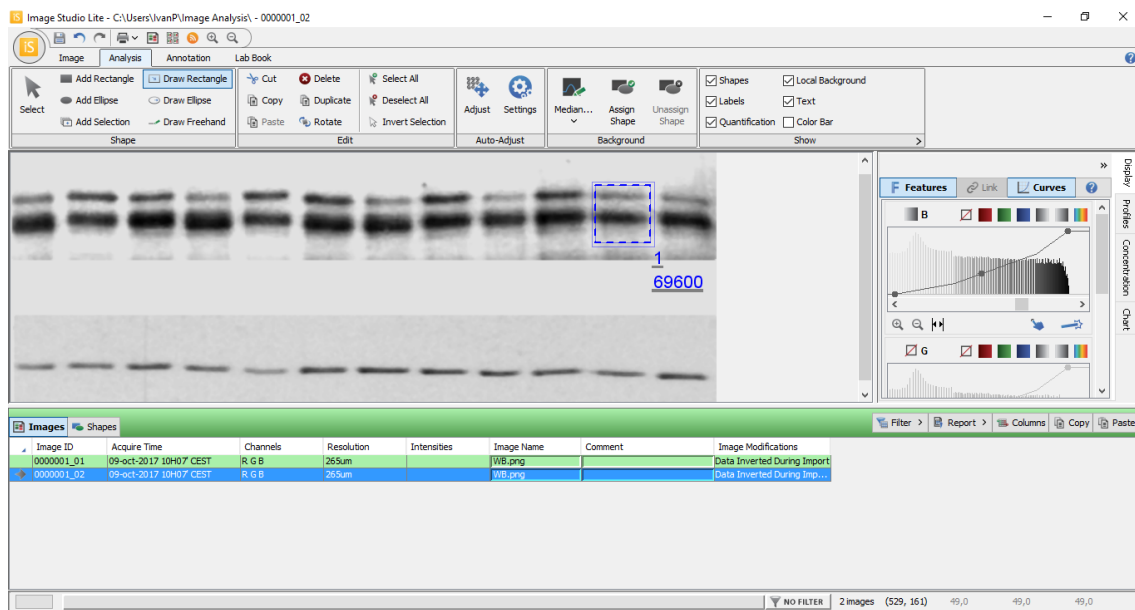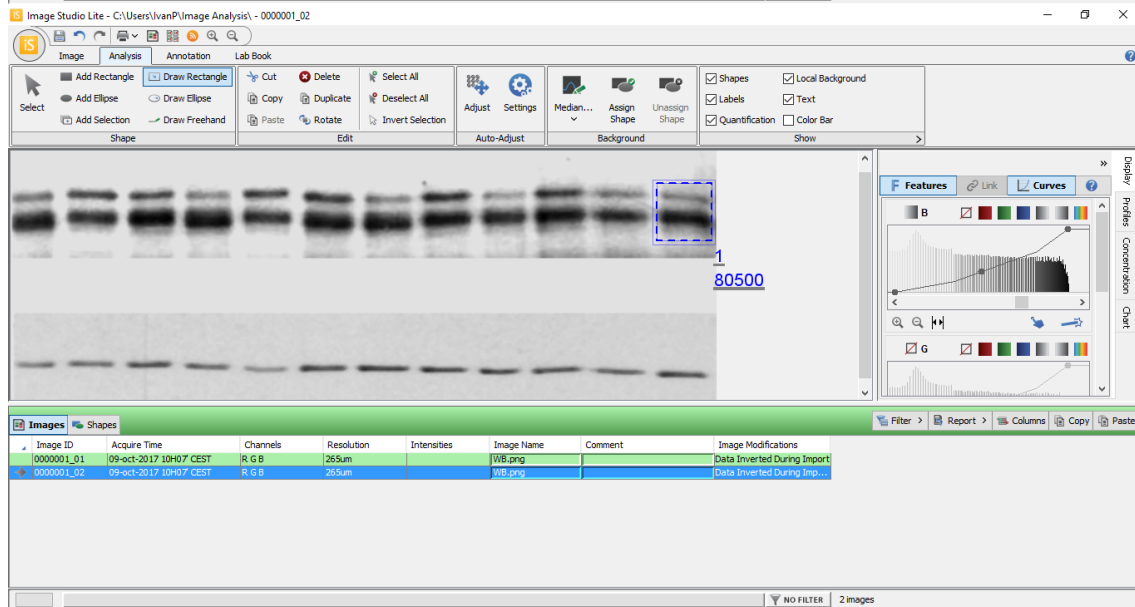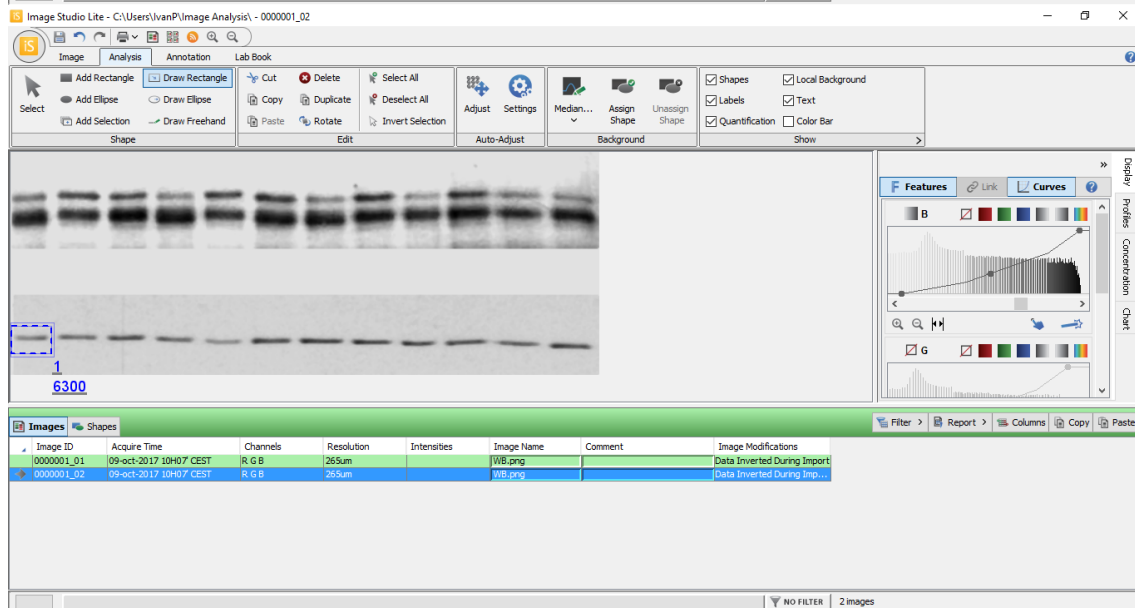

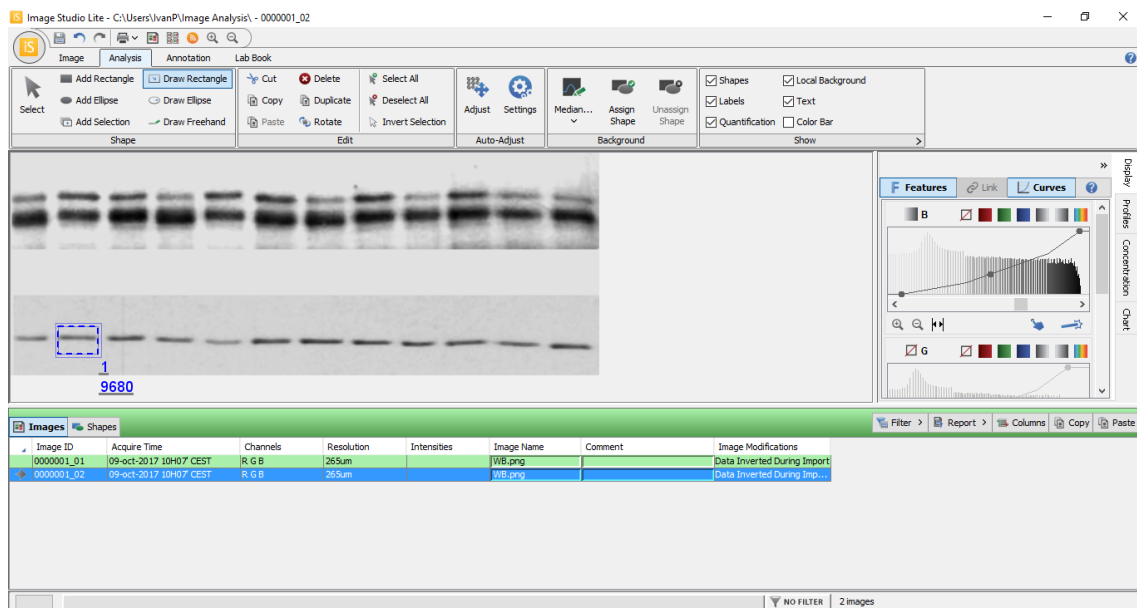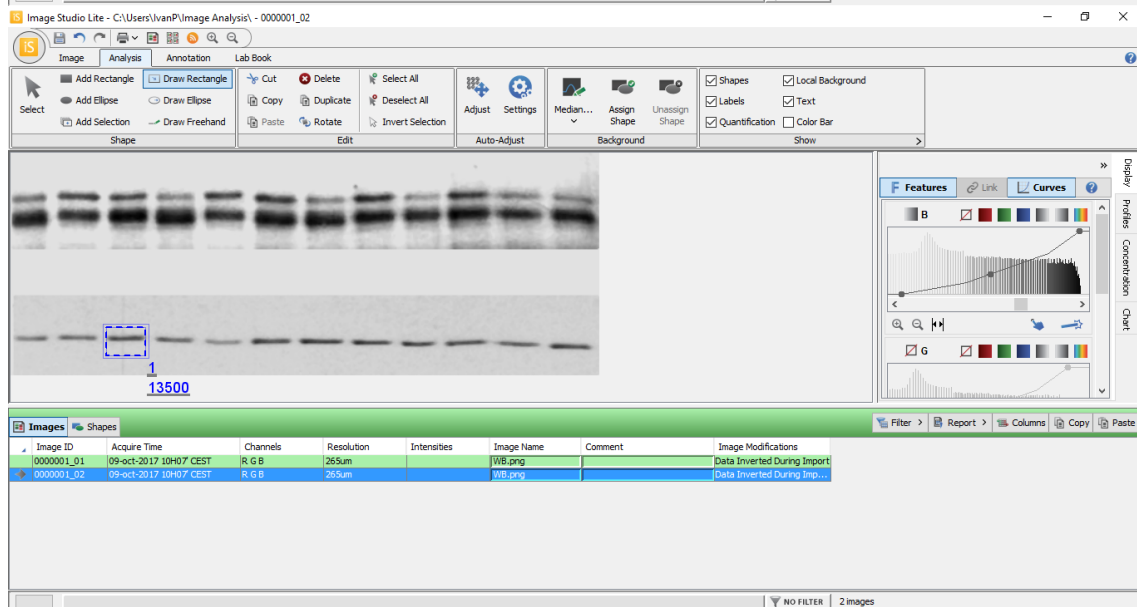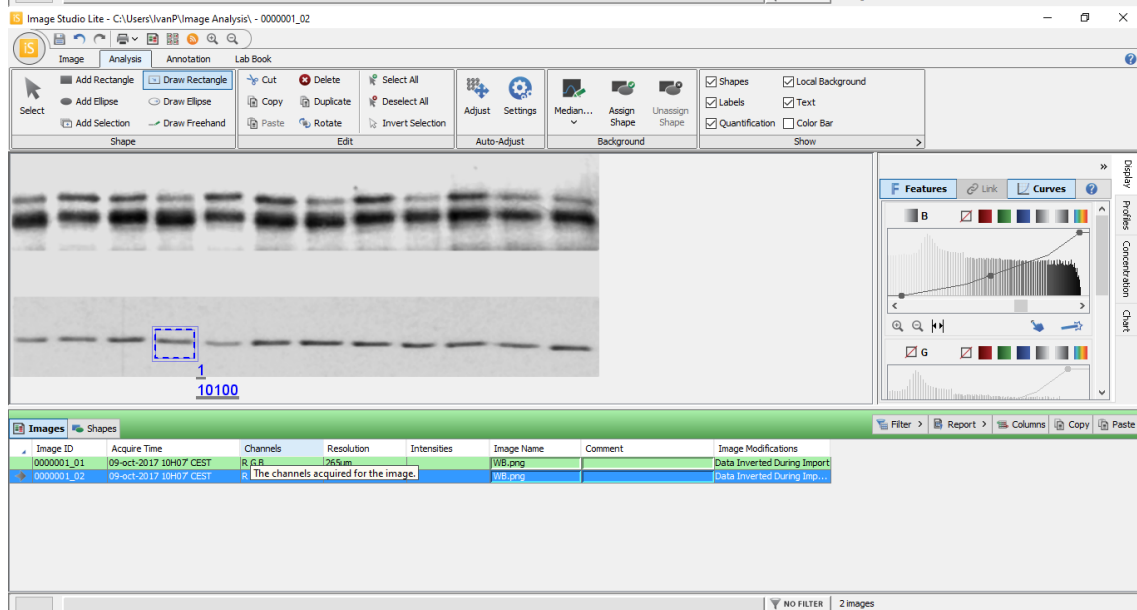

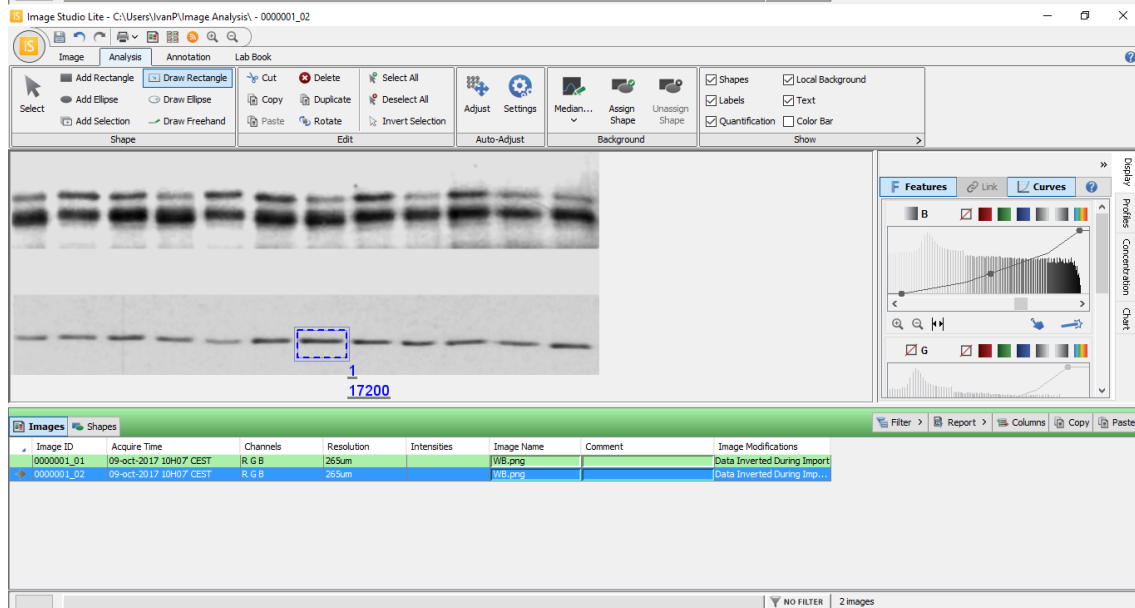

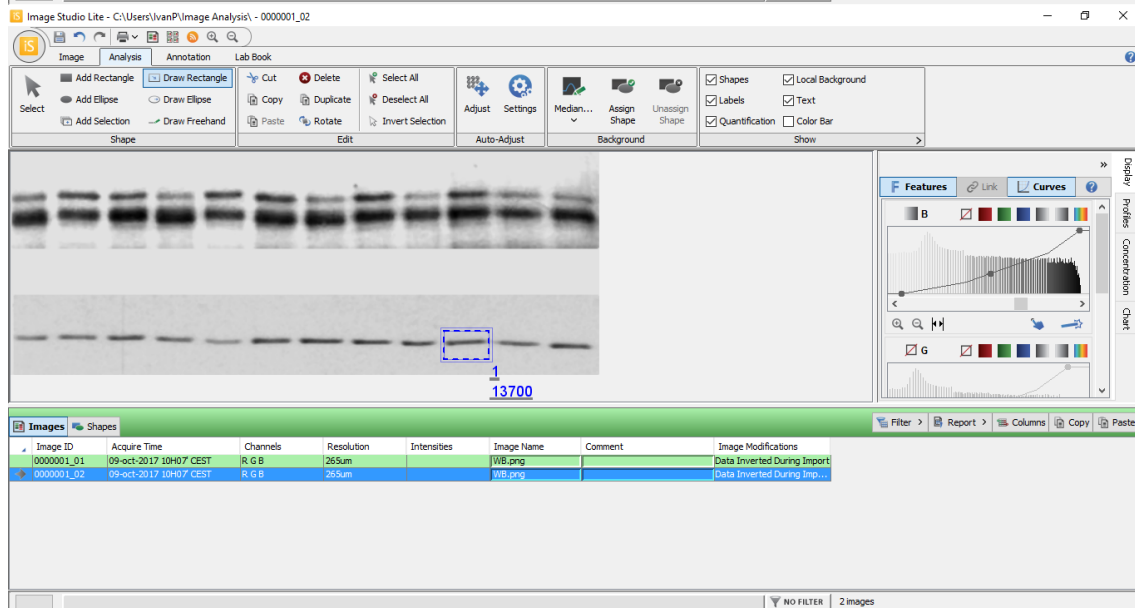

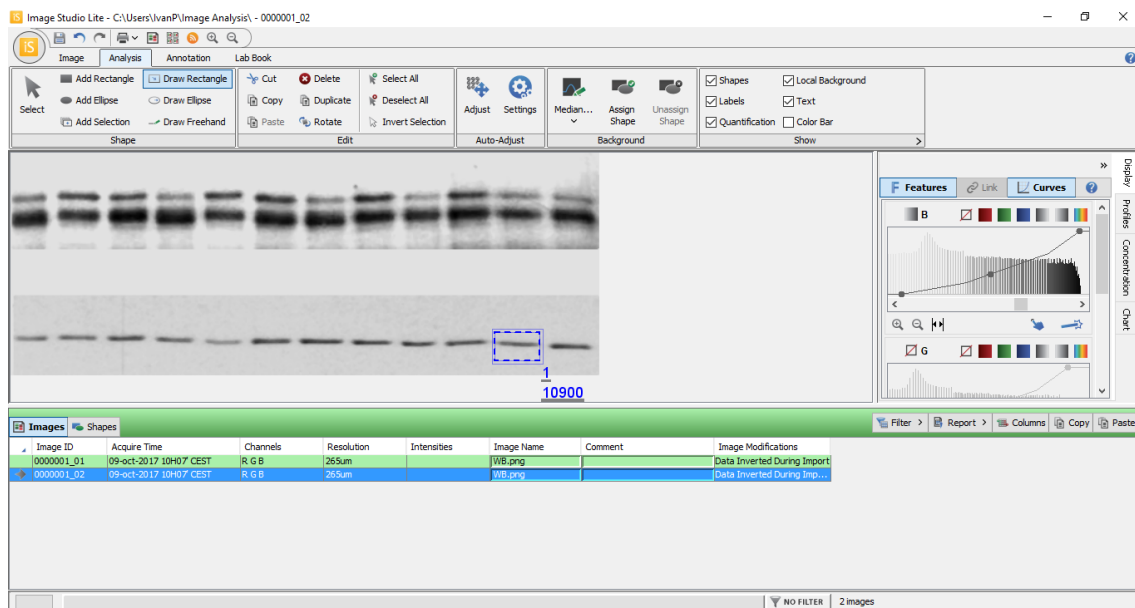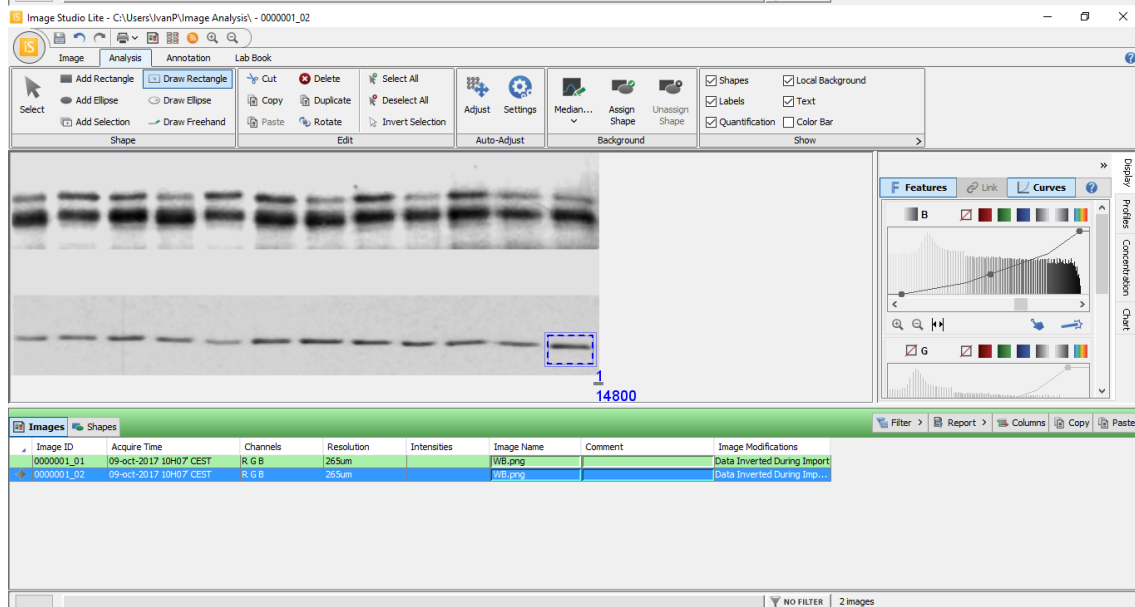

Supplement: Supplementary file 1 — Supplementary Material S1 [file 41431_2019_414_MOESM1_ESM.pdf]
